# Altered profile of glycosylated proteins in serum samples obtained from patients with Hashimoto′s thyroiditis following depletion of highly abundant proteins

**DOI:** 10.3389/fimmu.2023.1182842

**Published:** 2023-06-30

**Authors:** Yaozheng Xu, Jiawen Huo, Ruili Nie, Lili Ge, Chonghong Xie, Yuan Meng, Jianhua Liu, Lina Wu, Xiaosong Qin

**Affiliations:** ^1^ Department of Laboratory Medicine, Shengjing Hospital of China Medical University, Shenyang, China; ^2^ Liaoning Clinical Research Center for Laboratory Medicine, Shenyang, China

**Keywords:** Hashimoto′s thyroiditis, lectin microarray, glycosylation, VVA, LTF, MASP-1

## Abstract

**Objectives:**

Hashimoto’s thyroiditis (HT) is one of the most common autoimmune disorders; however, its underlying pathological mechanisms remain unclear. Although aberrant glycosylation has been implicated in the N-glycome of immunoglobulin G (IgG), changes in serum proteins have not been comprehensively characterized. This study aimed to investigate glycosylation profiles in serum samples depleted of highly abundant proteins from patients with HT and propose the potential functions of glycoproteins for further studies on the pathological mechanisms of HT.

**Methods:**

A lectin microarray containing 70 lectins was used to detect and analyze glycosylation of serum proteins using serum samples (N=27 HT; N=26 healthy control [HC]) depleted of abundant proteins. Significant differences in glycosylation status between HT patients and the HC group were verified using lectin blot analysis. A lectin-based pull-down assay combined with mass spectrometry was used to investigate potential glycoproteins combined with differentially present lectins, and an enzyme-linked immunosorbent assay (ELISA) was used to identify the expression of targeted glycoproteins in 131 patients with papillary thyroid carcinoma (PTC), 131 patients with benign thyroid nodules (BTN) patients, 130 patients with HT, and 128 HCs.

**Results:**

Compared with the HC group, the majority of the lectin binding signals in HT group were weakened, while the *Vicia villosa* agglutinin (VVA) binding signal was increased. The difference in VVA binding signals verified by lectin blotting was consistent with the results of the lectin microarray. A total of 113 potential VVA-binding glycoproteins were identified by mass spectrometry and classified by gene ontology (GO) and Kyoto encyclopedia of genes and genomes (KEGG) analyses. Using ELISA, we confirmed that lactoferrin (LTF) and mannan-binding lectin-associated serine protease 1 (MASP-1) levels were elevated in the serum of patients with HT and PTC.

**Conclusion:**

Following depletion of abundant proteins, remaining serum proteins in HT patients exhibited lower glycosylation levels than those observed in HCs. An increased level of potential VVA-binding glycoproteins may play an important role in HT development. LTF and MASP-1 expression was significantly higher in the serum of HT and PTC patients, providing novel insight into HT and PTC.

## Introduction

Hashimoto’s thyroiditis (HT) is the most common autoimmune thyroid disorder and is characterized by lymphocyte infiltration of the parenchyma and the presence of antibodies specific to thyroid antigens (1, 2). Currently, the prevalence of HT is 0.3–1.5 cases per 1000 individuals, and women are at least eight times more likely to develop HT than men (3, 4). HT is the leading cause of hypothyroidism, which frequently triggers local and systemic manifestations in the skin and cardiovascular system (4–6). Although HT is frequently observed in thyroid glands resected for a neoplastic process, to date, the association between HT and papillary thyroid carcinoma (PTC) remains unclear (7–10). Anti-thyroglobulin (anti-Tg) antibodies and anti-thyroperoxidase (anti-TPO) antibodies are considered two of the best serological markers for HT diagnosis, representing a more initial immune response and a later adaptive immune response, respectively (11). In addition to the functional alteration of B cells, T cell dysfunction is associated with the breakdown of immune homeostasis in thyroid tissue (12, 13). Several genetic, epigenetic, and environmental factors that can trigger an autoimmune response have been identified; however, the exact pathogenic mechanisms underlying HT remain unknown and warrant further study (14).

Glycosylation is one of the most complex post-translational modifications and is involved in many critical biological processes, such as protein folding and stability, cell growth, and cellular interactions (15–17). Changes in protein glycosylation are associated with the pathogenesis of diseases, such as cancer, infections, and autoimmune diseases (18, 19). Many studies have demonstrated the importance of glycans in regulating humoral and cellular immune responses, including immunoglobulin function (20, 21), T-cell development, and differentiation (22). Lectin microarray is a high-sensitivity and high-throughput glycosylation analysis technology that has been applied for biomarker identification and determining glycosylation profile, especially in autoimmune diseases and cancers (23, 24).

Glycosylation of thyroid proteins plays an important role in hormone synthesis, thyroid-stimulating hormone (TSH) activity and Tg transport (25, 26). Owing to the reflex of pathological conditions of autoimmunity and the high sensitivity to medicines applied in disease therapy, the N-glycome of immunoglobulin G (IgG) is one of the best-studied glycoproteins (27). The sugar composition of IgG Fc N-glycans determines the induction of pro- and anti-inflammatory signals (28), as well as antibody-dependent cell-mediated cytotoxicity (ADCC) and complement-dependent cytotoxicity (CDC) involved in the destruction of thyroid tissue in HT (29, 30). Decreased core fucosylation of the entire IgG N-glycan pool is currently used as a serum glycomarker of HT (31). The reduced core fucosylation of IgG is inversely related to anti-TPO level (32); however, fucosylation and sialylation of anti-Tg IgG are elevated in HT (31). A significant decreased *Lens culinaris* agglutinin (LCA) staining and increased *Maackia amurensis* II lectin (MAL-II) reaction were reported in IgG-depleted serum of HT, which resulted from the reduction of α1,6-linked core fucose and elevated level of α2,3-sialylation, respectively (33).

Several studies have provided evidence that HT is a risk factor for thyroid cancer (34). Similarly, thyroid carcinogenesis is accompanied by changes in sialylation and fucosylation in addition to O-GlcNAcylation (32). Compared with patients with PTC, the serum of HT patients has significantly lower core fucose content and a decreasing trend of Tg antibody sialylation (35). In addition, changes in IgG N-glycans have been demonstrated in other autoimmune diseases such as rheumatoid arthritis (RA) and systemic lupus erythematosus (SLE) (36, 37). IgG galactosylation is decreased in patients with RA (38), and a higher content of mannose (Man) and galactose (Gal) on α2-macroglobulin has been identified in patients with SLE (39).

So far, research on glycosylation has primarily focused on the diversification of the IgG glycome. However, deletion of highly abundant proteins enables the detection of signals from glycoproteins at lower concentrations and obtaining more reliable and accurate results (31). In this study, we employed a lectin microarray to examine the glycosylation levels of proteins in sera from HT patients and healthy controls (HCs), which were depleted of the top 14 abundant proteins. In addition, we used mass spectrometry and ELISA to investigate the differences in glycoproteins among various thyroid diseases to provide new insights into the pathogenic mechanisms underlying HT.

## Methods

### Serum samples

All serum samples used in the study were collected at Shengjing Hospital of China Medical University between 2018 and 2021. A total of 53 serum samples collected from 27 patients with HT and 26 healthy controls (HCs) were used for lectin microarray analysis. The characteristics of the study sample are listed in **Table 1**. For the lectin blot analysis, seven HT patients and seven HCs were randomly chosen from the lectin microarray analysis cohort. The patient characteristics of these samples are listed in **Table 2**. In addition, samples was collected from 131 patients with PTC, 131 patients with benign thyroid nodules (BTN), 130 patients with HT, and 128 HCs for enzyme-linked immunosorbent assay (ELISA). The patient characteristics of this cohort are listed in **Table 3**. There were no significant differences in the sex ratio or average age of the participants between the two groups. In the HT group, serum samples were collected from hospitalized patients who did not have goiter or other autoimmune diseases. Patients with PTC were diagnosed pathologically after other thyroid diseases were ruled out, whereas patients with BTN included those with benign thyroid tumors and nodular goiters. None of the participants in the HC group had autoimmune diseases, infections, cancers, or any severe comorbidities. The serum samples were obtained after centrifugation, transferred to individual microtubes, and stored at –80°C until use.

**Table 1:** Clinical characteristics of patients for lectin microarray.

	HC	HT
n	26	27
Male (%)	1 (3.8)	1 (3.7)
Age (years)	35 ± 7.3	34.3 ± 7.4
TSH (μIU\mL)	1.8 ± 0.6	2.1 ± 1.7
FT3 (pmol\L)	4.3 ± 0.6	6.3 ± 5.7
FT4 (pmol\L)	12.5 ± 0.8	16.2 ± 8.1*
TPOAb (IU\mL)	1.8 ± 1.4	293.8 ± 375.9***
TgAb (IU\mL)	1.8 ± 1.4	171.9 ± 153.8***

Characteristics of healthy control (HC) group and Hashimoto′s thyroiditis (HT) group. Data are expressed as means ± SD. * p <0.05 vs. healthy controls group, *** p <0.005 vs. healthy controls group. TSH, thyroid-stimulating hormone; FT3, free triiodothyronine; FT4, free thyroxine; TPOAb, anti-thyroperoxidase; TgAb, thyroglobulin.

**Table 2:** Clinical characteristics of patients for lectin blot.

	HC	HT
n	7	7
Male (%)	1 (14.3)	1 (14.3)
Age (years)	34.6 ± 5.7	35.9± 6.6
TSH (μIU\mL)	1.6 ± 0.5	3 ± 0.8**
FT3 (pmol\L)	4.2 ± 0.3	4.4 ± 0.3
FT4 (pmol\L)	12.3 ± 1.1	12.4 ± 0.9
TPOAb (IU\mL)	2.2 ± 0.8	428.6 ± 446.6*
TgAb (IU\mL)	1.4 ± 0.7	175.2± 122.4**

Characteristics of healthy control (HC) group and Hashimoto′s thyroiditis (HT) group. Data are expressed as means ± SD. * p <0.05 vs. healthy controls group, ** p <0.01 vs. healthy controls group.

**Table 3:** Clinical characteristics of patients for ELISA.

	HC	HT	PTC	BTN
n	128	130	131	131
Male (%)	22 (17.2)	20 (15.4)	19 (14.5)	22 (16.8)
Age (years)	46.0 ± 9.3	47.6 ± 10.5	46.8 ± 9.2	47.1 ± 9.5
TSH (μIU\mL)	1.8 ± 0.8	5.7 ± 15.2**	1.9 ± 1.3	1.7 ± 1.2
FT3 (pmol\L)	4.5 ± 0.4	4.4 ± 1.7	4.3 ± 0.6***	4.3 ± 0.6***
FT4 (pmol\L)	13.4 ± 1.2	12.9 ± 3.3	13.5 ± 2.2	13.4 ± 1.8
TPOAb (IU\mL)	1.2 ± 1.3	509.4 ± 422.3***	49.9 ± 197.1***	68.1 ± 236.4**
TgAb (IU\mL)	1.4 ± 0.8	224.4 ± 298.5***	57.0 ± 179.3***	39.5 ± 144.5**

Characteristics of healthy control (HC) group, Hashimoto′s thyroiditis (HT) group, papillary thyroid carcinoma (PTC) group and benign thyroid nodules (BTN) group. Data are expressed as means ± SD. ** p <0.01 vs. healthy controls group, *** p <0.005 vs. healthy controls group.

### Lectin microarray

A commercial lectin microarray (Raybiotech, Guangzhou, China) containing 70 lectins was used to investigate the glycopatterns of serum that were depleted of high-abundance proteins, according to the manufacturer’s protocols. First, each serum sample (10 μL) was depleted using the High Select Top 14 Abundant Protein Depletion Mini Spin (Thermo Fisher Scientific, Waltham, MA) and labelled. Then the lectin microarrays were removed from -20°C storage and incubated at room temperature for 1 h. Microarrays were incubated with blocking buffer at room temperature for 30 min. After washing and drying, 100 μL sample (1:30 dilution) was applied to the microarray and incubated overnight at 4°C. The microarrays were washed five times with wash buffer I and twice with wash buffer II, followed by incubation with 5 mL secondary antibody in the dark at room temperature for 1 h. After five washes with wash buffer I and two washes with wash buffer II, the microarrays were dried and scanned using an Axon GenePix 4000 B Microarray Scanner at a wavelength of 532 nm.

The microarray images were analyzed by GenePix Pro 6.0 software (Molecular Devices, Sunnyvale, CA). The signal-to-noise ratio (S/N) of each lectin spot was calculated, and the S/N data were normalized between different blocks. For differential level, lectin must meet the following two conditions: (a) fold change (HT/HC) >1.2 or <0.83, and (b) P value <0.05.

### Lectin blot analysis

To validate the results of the lectin microarray analysis, seven HT patients and seven HC were randomly selected from the lectin microarray analysis cohort. The protein concentrations of the serum samples were quantified using the BCA assay (CWbiotech, Beijing, China). Briefly, to verify the binding of serum that was depleted of high abundance proteins or not to lectins, 7 μg serum protein sample containing high abundance proteins was separated by 10% sodium dodecyl sulfate–polyacrylamide gel electrophoresis (SDS–PAGE). The separated proteins were transferred to PVDF membranes (Millipore, Billerica, MA, USA) while the duplicate gels were stained with Coomassie brilliant blue. The membranes were blocked with 1% BSA in phosphate buffered saline with 0.1% Tween 20 (PBS-T) at room temperature for 1 h and probed with biotinylated lectins, including *Vicia villosa* lectin (VVA), *Maackia amurensis* lectin (MAA), *Solanum tuberosum* lectin (STL), *Sambucus nigra I* lectin (SNA-I) and *Datura stramonium* lectin (DSA) (1:400; Vector Laboratories Inc., US) at 4°C overnight in the dark. After three washes with PBS-T, the membranes were incubated with HRP-conjugated streptavidin (Yeasen, Shanghai, China). The reactive signals were detected using a Tanon 550 Multi Fluorescence Signal System (Tanon, Shanghai, China). ImageJ software was used for signal intensity analysis.

### Glycoprotein pull-down assay

For identifying suspected glycoproteins, a lectin-based pull-down assay was conducted using Pierce spin columns (Thermo Fisher Scientific, Waltham, MA). In brief, the columns were incubated with 300 μL biotin labelled VVA (1 μg/mL) at 4°C for 1 h and blocked at room temperature for 5 min. Then, 300 μL samples of diluted serum from 10 HT patients and 10 HCs were incubated at 4°C for 1 h. After three washes, the bead-bound glycoproteins were reduced by 10 mM DTT at 37°C and alkylated using 25 mM iodoacetamide for 20 min in the dark. Trypsin digestion was performed at 37°C and the peptides were extracted using NH_4_HCO_3_ buffer. After removing the salt with acetonitrile and drying, the samples were subjected to mass spectrometry.

### Mass spectrometry

Proteins were analyzed qualitatively and quantitatively using an Orbitrap Fusion Lumos mass spectrometer (Thermo Scientific, USA) coupled with an EASY-nLC 1000 liquid chromatography system (Thermo Scientific, USA). The vacuum-dried samples were reconstituted with 0.1% formic acid (FA) and separated on a 75 μm I.D. × 25 cm C18 homemade analytical column (C18, 1.9 μm, 120 Å, Maisch GmbH, Germany) with a mobile solution flow rate of 200 nL/min. The gradient elution program was as follows: 2-5% solvent B (100% acetonitrile and 0.1% formic acid) for 3 min, 5-8% solvent B for 20 min, 8-16% solvent B for 19 min, 16-28% solvent B for 5 min, 28-80% solvent B for 1 min, and 80% solvent B for 12 min. Data were acquired using full scans (m/z 350–1800) at a mass resolution of 60,000 (FWHM). For MS2, the normalized automatic gain control (AGC) target was 1E+06 with a resolution of 15,000 and a maximum injection time of 22 ms. The precursor ions were fragmented in the high-energy collision dissociation (HCD) cell at a normalized collision energy (NCE) of 30%.

The raw files obtained from the Orbitrap Fusion Lumos mass spectrometer were analyzed using Proteome Discoverer 2.4 (Thermo Scientific, USA), and Swiss-prot human database. The search conditions were as follows: fixed modification was set as carbamidomethyl (C) and variable modification was set as methionine oxidation (M) and protein N-terminal acetylation (acetyl). Trypsin digestion was performed to allow for two missing sites. The maximum mass errors of the mother and daughter ions were set to 20 ppm and 0.5 Da, respectively. The false-positive rate of the peptide segment (FDR) was <0.01.

### ELISA

The Human MASP1 ELISA Kit and Human LTF ELISA Kit (Mlbio, Shanghai, China) were used to estimate the antigen titers of participants in the PTC, BTN, HT, and HC groups, according to the manufacturer’s protocols. Each well of the microtiter plate was incubated with a mixture of 50 μL human serum or standard and 100 μL enzyme conjugate for 1 h at 37°C. After five washes, 100 μL substrate solution was added and incubated in the dark at room temperature for 15 min. The reaction was stopped by adding 50 μL stop solution, and the absorbance at 450 nm was read immediately.

### Statistical analysis

GraphPad Prism 9 was used to perform all statistical analyses. A Student’s *t*-test was performed to calculate statistically significant differences in results among the PTC, BTN, HT and HC groups, and results with *P <*0.05 were considered statistically significant.

## Results

### Serum glycosylation pattern determination in patients with HT using lectin microarray analysis

Serum samples from 27 HT patients and 26 HCs that were depleted of the top 14 most abundant protein were analyzed using a lectin microarray. As shown in **Table 1**, the mean age of the HT group was 34.3 years and only 3.7% of the patients were male. The mean age of the HC group was 35 years, and 3.8% of the patients were male. There were no significant differences in the average age and sex ratios between the two groups. The levels of TPOAb and TgAb were significantly higher in the HT group than those for HC group. There were no significant differences in TSH and FT3 levels between these two groups, while a slight difference was noted in FT4 level.

**Figure 1:**
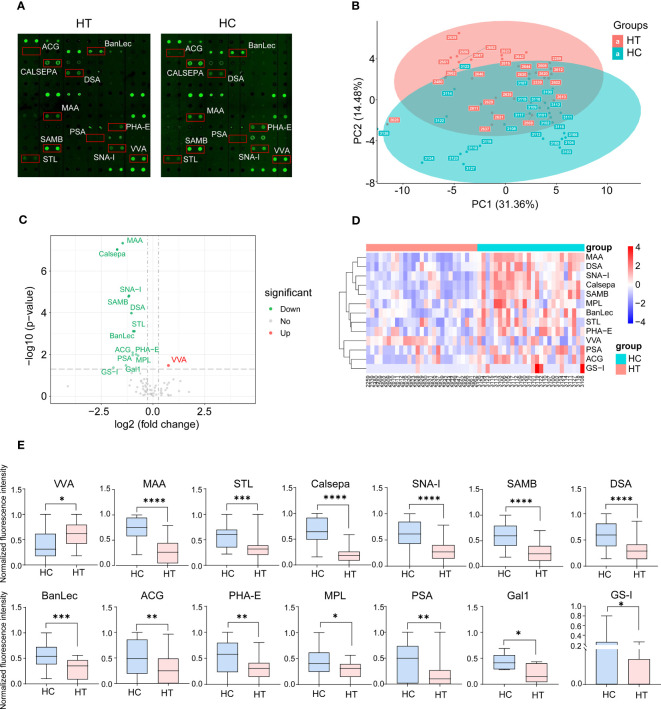
Lectin array profiling of serum samples from Hashimoto′s thyroiditis (HT) patients (n=27) and healthy control (HC) (n=26). **(A)** Representative results of the array profiling for HT (left) and HC (right) groups. Red boxes indicate the position of lectins with the significant difference between groups. **(B)** Principal component analysis (PCA) of the mean normalized signals obtained from lectin microarray. A scatter plot for principal component (PC) 1 and 2 is shown. Each dot represents the sample derived from an individual person. Different groups are indicated by color. **(C)** Volcano plot of log2 fold change (x axis) in lectin binding signals of HT samples *vs*. HC samples against the significance of change shown as the −log10 p-value (y axis). Red and green dots indicate lectins with up-regulated and down-regulated binding signals, respectively. **(D)** Heat map and hierarchical clustering of lectin microarray signals. Each column represents samples from an individual person. Thirteen lectins are shown on rows. **(E)** The binding signal of 14 lectins shows a significant difference between groups. *p <0.05, **p <0.01, ***p <0.005 and ****p <0.001. VVA, *Vicia villosa* lectin; MAA, *Maackia amurensis* lectin; STL, *Solanum tuberosum* lectin; Calsepa, *Calystegia sepium* lectin; SNA-I, *Sambucus nigra I* lectin; SAMB, *Sambucus Sieboldiana* lectin; DSA, *Datura stramonium* lectin; BanLec, *Musa acuminata* lectin; ACG, *Agrocybe cylindracea* lectin; PHA-E, *Phaseolus vulgaris Erythroagglutinin*; MPL, *Maclura pomifera* lectin; PSA, *Pisum sativum* lectin; Gal1, *Human galectin1* lectin; GS-I, *Griffonia simplicifolia I* lectin.

The layout of the lectin microarray and representative profiling images are shown in **Figure 1A** and **Supplementary Figure 1**. These two groups showed a separation trend according to principal component analysis (PCA) (**Figure 1B**). Fourteen of the 70 lectins showed differential signal intensity between the HT and HC groups (p < 0.05). Thirteen lectin-binding signals were weakened in HT patients, whereas one binding signal was enhanced (**Figures 1C, D**). Subsequently, VVA, MAA, and STL were selected for further validation. Compared with the HC group, the VVA binding signal was increased (fold change = 1.68); however, the MAA (fold change = 0.36), STL (fold change = 0.54), Calsepa (fold change = 0.30), SNA-I (fold change = 0.45), SAMB (fold change = 0.44), DSA (fold change = 0.48), BanLec (fold change = 0.52), ACG (fold change = 0.51), PHA-E (fold change = 0.57), PSA (fold change = 0.51), MPL (fold change = 0.61), Gal1 (fold change = 0.40) and GS-I (fold change = 0.27) binding signals were decreased (**Figure 1E**). However, these varied lectin signals did not correlate with TSH levels using Pearson correlation analysis (**Supplementary Table 2**).

### Validation of glycosylation changes of HT serum using lectin blot analysis

Serum samples from seven HT patients and seven HCs that were depleted of the top 14 most abundant proteins were randomly selected from the lectin microarray cohort to verify the lectin blot results. As shown in **Table 2**, the mean age of the HT group was 35.9 years and 14.3% of the patients were male. The mean age of the HC group was 34.6 years, and 14.3% of the patients were male. There were no significant differences in the average age and sex ratios between the two groups. The levels of TSH, TPOAb and TgAb were significantly higher in the HT group than those for HC group.

**Figure 2:**
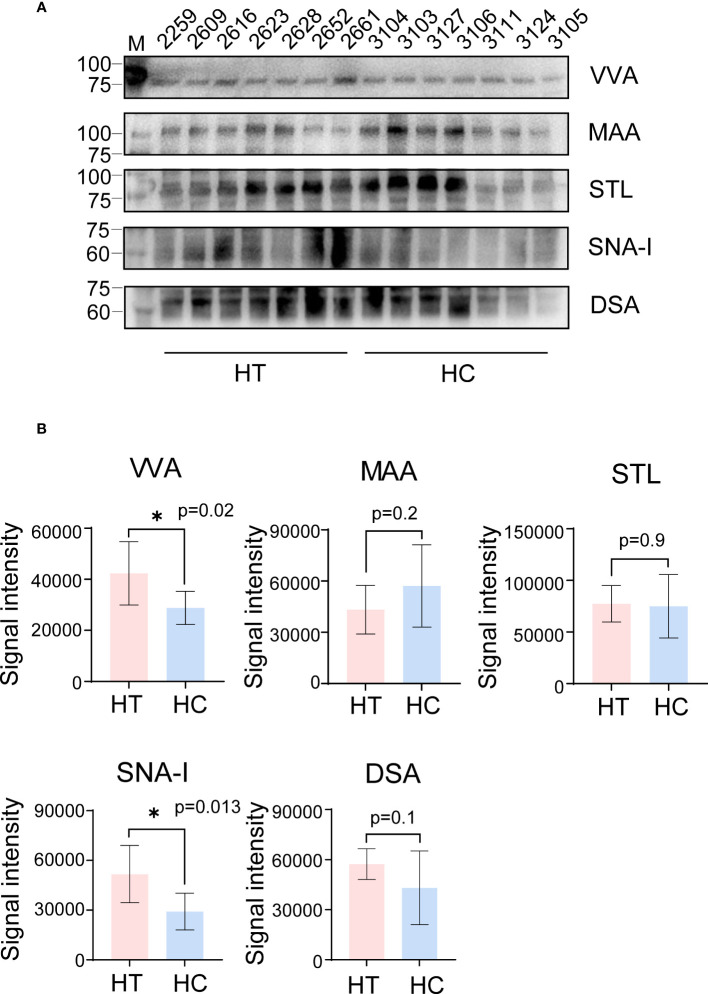
Lectin blot verification of a small cohort of serum samples profiled by lectin array. **(A)** The VVA, MAA, STL, SNA-I and DSA blot using serum samples obtained from the HT (left 2-8 column) and HC group (left 9-15 column). The number above each column indicates the corresponding sample. VVA and MAA bands are shown at ~75 KDa and 100 KDa, respectively. Band for STL blot was between 75 to 100 KDa on SDS-PAGE. SNA-I and DSA bands are shown at ~60 KDa. **(B)** The signal intensity analysis of bands from VVA, MAA, STL, SNA-I and DSA blot. All error bars indicate SD. *p <0.05.

Compared with the HC group, VVA binding increased at a molecular weight of ~75 KDa in the HT group, indicating that some proteins of ~75 kDa were over-sialylated or overexpressed in the serum of HT patients (**Figure 2A**; **Supplementary Figure 2**). In addition, the increase in the VVA binding signal (fold change = 1.47) was consistent with the array profiling (**Figure 2B**). Although the decrease for MAA was not significant, a tendency was observed, consistent with the array profiling. However, the tendency for the SNA-I and DSA binding signals was contrary to the array profiling. There was no difference in the STL results between the two groups, so we chose VVA for further studies. To explore VVA binding in HT serum containing abundant protein, we repeated the lectin blot analysis using the same serum samples which were not depleted of abundant proteins and achieved the same results as those for the VVA blot (**Supplementary Figure 3**).

### Enrichment analysis of glycoproteins combined with VVA using mass spectrometry

**Figure 3:**
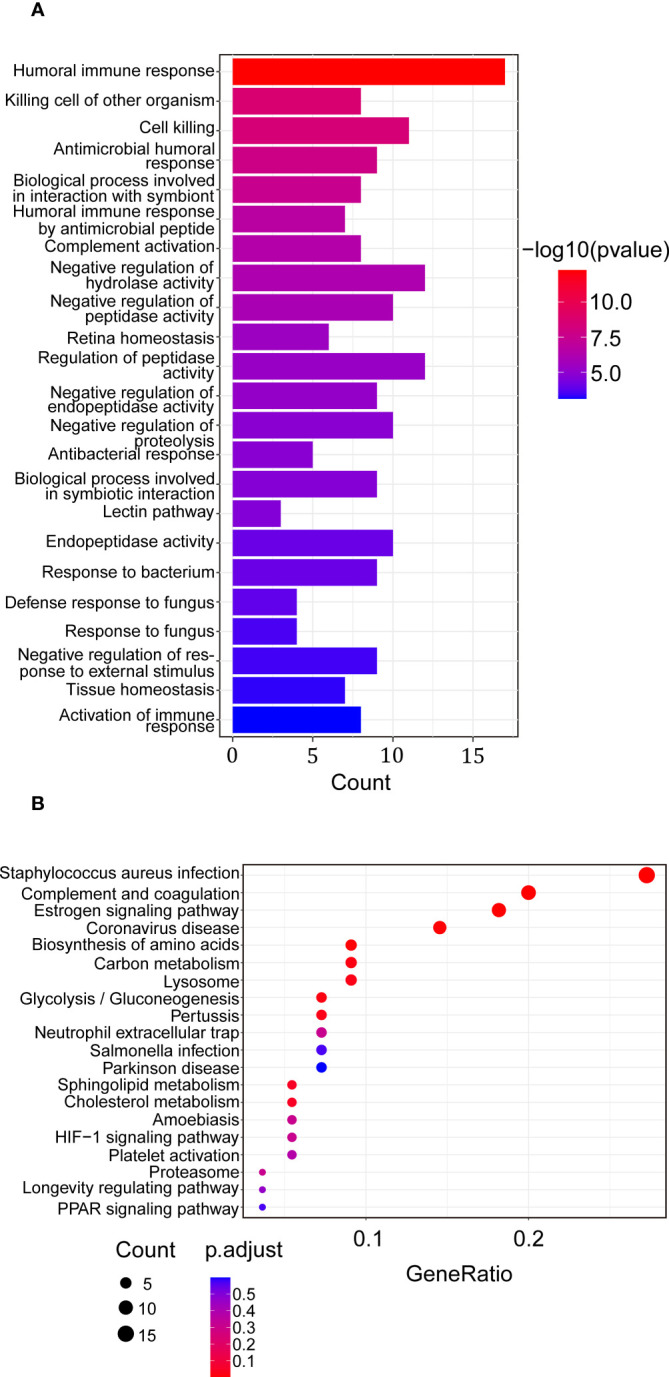
Enrichment analysis of proteins up-regulated in binding of VVA between the HT *vs*. HC group. **(A)** Biological process of GO analysis. The horizontal coordinate represents the genes count, the vertical coordinate shows the GO terms, the column color indicates -log10 (p-value). **(B)** KEGG analysis. The horizontal coordinate represents the gene ratio, the vertical coordinate represents the terms, the node color represents p.adjust, and the node size represents the number of genes enriched in the pathway.

To study the proteins combined with VVA, a lectin-based pull-down assay was performed and VVA was used as bait during incubation of serum sample obtained from HT patients and HCs. The samples were then subjected to mass spectrometry analysis. We identified 113 potential VVA-binding glycoproteins classified by gene ontology (GO) and Kyoto encyclopedia of genes and genomes (KEGG) analyses (**Supplementary Table 1**). Biological process identified by GO analysis of the 97 glycoproteins showed that potential VVA-binding glycoproteins were significantly enriched for humoral immune response and cell killing. Interestingly, some glycoproteins were enhanced for complement activation and the lectin pathway (**Figure 3A**). In the KEGG pathway analysis of 55 VVA-binding glycoproteins, the majority participated in the Staphylococcus aureus infection, complement, coagulation, and estrogen signaling pathways. A few glycoproteins were also found to play roles in gluconeogenesis and platelet activation (**Figure 3B**).

### Alteration of LTF and MASP-1 in thyroid diseases

Due to the clear signal near 75 KDa in the lectin blot analysis, serum samples of 131 PTC patients, 131 BTN patients, 130 HT patients, and 128 HCs were used to detect the concentrations of LTF (78.1 KDa) and MASP-1 (79.2 KDa) by ELISA. As shown in **Table 3**, the mean age of the PTC group was 46.8 years and 14.5% were male, the mean age of the BTN group was 47.1 years and 16.8% were male, the mean age of the HT group was 47.6 years and 15.4% were male, and the mean age of the HC group was 46 years and 17.2% were male. There were no significant differences in the average age and sex ratio between the groups. The levels of TPOAb and TgAb in patients with PTC, BTN, and HT were significantly higher than those in the HC group, especially in the HT group.

**Figure 4:**
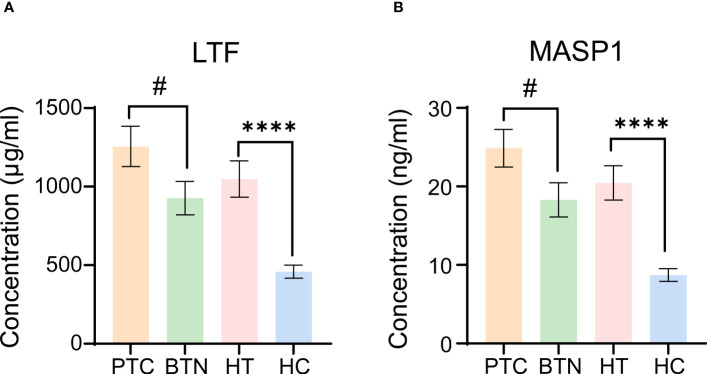
Evaluation of LTF and MASP-1 levels in serum samples from different groups. **(A, B)** LTF **(A)** and MASP-1 **(B)** levels were measured by ELISA. All error bars indicate SEM. # p <0.05, ****p <0.001. PTC, papillary thyroid carcinoma; BTN, benign thyroid nodules.

LTF concentration in the HT group was significantly higher than that in the HC group. Correspondingly, patients with PTC had higher LTF than those in the BTN and HT groups (**Figure 4A**). Similar to LTF, the concentration of MASP-1 was elevated not only in the HT group but also in the PTC and BTN groups compared with the HC group. There was an evident difference between the PTC and BTN groups (**Figure 4B**). However, the concentrations of LTF and MASP-1 in the HT and PTC samples did not correlate with TSH levels using Pearson correlation analysis (**Supplementary Tables 3, 4**).

## Discussion

HT is one of the most common autoimmune diseases; however, its exact etiology has not been fully elucidated. Lectin microarray analysis, first introduced in 2005, offers advantages such as rapid profiling, high throughput and high sensitivity (40, 41). Recently, lectin microarrays have been widely used to explore glycosylation in cancer and autoimmune diseases. In the present study, we evaluated 27 HT and 26 HC serum samples that were depleted of the top 14 abundant proteins using lectin microarrays to comprehensively study the glycosylation status in HT. We found that most lectin signals weakened in addition to VVA in the 14 altered lectins. To our surprise, only the VVA binding signal was consistent with the array profiling among the five lectins verified using lectin blotting. We consider that this result can be mainly attributed to the complexity of the glycoproteins, especially in complex biological samples. However, inter-patient variability is high and diverse thyroid functions in different patients may contribute to the diversity in the lectin binding signals. In addition, the lectins used for the microarray and blotting analyses were prepared by different companies, which likely led to inconsistent results. Nonetheless, compared to other lectins, the changes in VVA binding signals in the serum of patients with HT were relatively stable and warrant further studies. VVA binding to CD8^+^ T cells may play an important role in augmenting immune responses in autoimmune diseases, such as RA and SLE (42, 43). In particular, CD8^+^ T cell counts are significantly increased and important to signaling pathways in HT (44). Therefore, VVA may be involved in the excessive immune response observed in HT. In our study, thirteen lectin-binding affinities were found weakened in patients with HT through lectin microarray analysis. We assume that this phenomenon is due to the decreased expression of glycosyltransferases. The expression of glycosyltransferases is the main factor influencing cell glycomes (45). Researchers have found reduced levels of FUT8, MGAT5, and ST6Gal1 gene transcripts in CD4+ T helper cells from patients with HT, which indicates lower core fucosylation and sialylation of glycoconjugates (46). Although serum and cell glycomes are diverse, the decreased expression of glycosyltransferases may be the key reason for the weakened lectin signals. Our lectin microarray analysis showed that the levels of two sialic acid-specific lectins, MAA and SNA-I, were reduced in the serum of patients with HT. A decrease in sialylation is a characteristic feature of malignant transformation in the thyroid gland (47). Although the association between HT and PTC is controversial, we hypothesize that sialylation may play important roles in the progression from health status to HT to PTC. Vanessa et al. (48) found that galectin-1 (Gal1) was markedly increased in thyroid carcinoma and could be a reliable diagnostic marker, while, in our study, Gal1 slightly decreased in the HT samples. We found that these fourteen varied lectin signals did not correlate with the TSH levels in HT using Pearson correlation analysis, which may be due to the normal thyroid function of patients with HT selected for lectin microarray analysis, as shown in **Table 1**.

Using GO analysis, we found that the potential VVA-binding glycoproteins were significantly enriched for humoral immune response and cell killing, as well as complement activation and the lectin pathway. Seventeen of the 97 glycoproteins, including LTF and MASP-1, are involved in the humoral immune response; 11 participate in cell killing, which is part of the cellular immune response; and 6 participate in both humoral and cellular immunity, which are closely interrelated and play key roles in HT development. Intrathyroidal B cells produce TgAb and TPOAb, some of which may act as antigen-presenting cells (APCs), thereby enhancing the autoimmune T-cell response. T helper 1 (Th1) cells are primarily involved in cellular immunity, while Th2 cells regulate humoral immunity in HT. Th17 cells secrete IL-17 that mediates tissue injury (49). Platelet factor 4 (PF4), one of the 6 glycoproteins mentioned above, has been shown to reduce subclinical hypothyroid autoimmune thyroiditis (50). A previous study reported that LTF, another of the 6 glycoproteins, was upregulated in HT patients (51). Complement pathway is overactivated in HT and PTC; all three pathways are activated in HT, whereas the alternative complement pathway and the mannose binding lectin (MBL) pathway are activated in PTC (52). In HT, C4 complement and all downstream components of the complement pathway are overexpressed in thyrocytes. In addition, C4 binds to TPOAb and mediates thyrocyte lysis (53). MASP-1 triggers activation of the lectin pathway and is altered in several autoimmune diseases, such as type 1 diabetes, but its role in HT has not yet been studied (54, 55).

In the present study, KEGG analysis revealed that potential VVA-binding proteins might be enriched in *S. aureus* infection, the estrogen signaling pathway, complement and coagulation, gluconeogenesis, and platelet activation. Increasing clinical evidence has shown associations between gut or skin microbiota and autoimmune diseases (56, 57). An increase in *S. aureus* skin colonization promotes inflammation in SLE and IgA deposition in glomerulonephritis (58, 59) and patients with RA are susceptible to *S. aureus* infections (60). Intestinal dysbiosis and bacterial overgrowth have been identified as factors that favor HT development and a thyroid–gut axis has been proposed (61). In addition, *S. aureus* has been found to evade immunity by altering cell-wall glycosylation (62). Thus, we infer that the glycosylation of *S. aureus* may play roles in HT development. The female-to-male rate ratio of autoimmune thyroid diseases (AITD) is reported at 4-6:1 in the general population (63). Estrogens influence lymphocyte proliferation and antibody production, and significantly contribute to the female predilection of AITD (64). Estrogen affects the production and activation of TPOAb and TgAb. In PTC, estrogens participate in the production of mutagenic molecules in thyroid cells as well as the proliferation and metastasis of tumor cells by regulating the thyrocyte enzymatic machinery and inflammatory processes (65, 66). Robert et al. (67) reported shorter prothrombin time ratio and activated partial thromboplastin time in HT patients, whereas euthyroid women with HT were characterized by abnormal coagulation and fibrinolysis. However, decreased ADP-induced platelet aggregation and a positive correlation between platelet count and FT4 levels have been identified in patients with HT (68). As a result, VVA increase resulting from enhanced N-acetylglucosamine plays an essential role in HT progression and is crucial for its underlying pathogenic mechanisms.

Finally, we found that LTF and MASP-1 levels were greatly increased in HT patients compared with those in HCs. Meanwhile, compared with patients with benign thyroid tumors and nodular goiters, an increase in LTF and MASP-1 was also observed in patients with PTC. LTF is vital for nonspecific immune responses and plays an important role in tumor progression (69). Chang et al. (51) reported LTF upregulation in HT, which is in accordance with our results; however, its downregulation in PTC is inconsistent with our results. This may be due to differences in the types of samples and patients enrolled. Chang et al. detected LTF expression in thyroid tissues, whereas our results were from the serum of patients with PTC. LTF is mainly secreted by epithelial cells in the mammary glands (70). Studies have reported that thyroid autoimmunity is an important cause and negative prognostic factor for breast cancer (71). A strong correlation between HT and breast cancer has been identified, with the possibility that women diagnosed with HT has a higher risk of developing breast cancer (72). As a result, LTF overexpression in serum may be induced by the mammary glands. Rujia et al. (73) reported similar results with lower LTF expression levels in tumor tissues and thyroid follicular epithelial cells. Studies have demonstrated that LTF methylation is significantly increased in tumor tissues and LTF level is closely related to immune activity. LTF plays a role in the differentiation of immature B cells into efficient antigen-presenting cells, potentiating adaptive recall responsiveness (74, 75). LTF possesses bi-antennary or multiple poly antennary glycans, whose activities toward immune regulation are dependent upon specific and varied patterns of glycosylation (76, 77). However, the role of LTF glycosylation in HT immunoregulation is worth investigating. To the best of our knowledge, the biological function of MASP-1 in autoimmune thyroiditis and thyroid carcinoma has not been studied. However, MASP-1 has been shown to play a crucial role in autoimmune diseases such as rheumatoid arthritis and type 1 diabetes (55, 78). MASP-1 activity is essential for autoimmune-associated inflammatory tissue injury mediated *via* activation of the alternative complement pathway (79). In addition, MASP-1 plays an essential role in the initiation of the lectin pathway of the complement system, which is a central effector arm of the immune system (80). However, we did not record any significant correlations between the levels of TSH with those of LTF or MASP-1, indicating that LTF and MASP-1 do not affect the thyroid function directly. Taken together, these results suggest that LTF and MASP-1 may participate in various pathways involved in the pathological mechanisms underlying HT and PTC.

Despite presenting some interesting findings, our study had some limitations. Owing to the single source and small sample size, it is necessary to use multi-center samples and expand the sample size to eliminate systematic errors for identifying more reliable differentially expressed lectins. Glycoproteins in HT patients with hyperthyroidism and hypothyroidism may have different functions; therefore, the serum samples could be grouped more extensively. Due to the significance of estrogen to autoimmune diseases, the menstruation status of patients and healthy controls should be included. To eliminate gender differences between the HT and PTC groups, the female-to-male ratio in the PTC group was higher than natural condition, which may have affected PTC research.

This is the first study to use lectin microarrays to analyze changes in serum depleted of highly abundant proteins and confirmed the increased signal intensity of the VVA lectin. We infer that glycoproteins may play important roles in the pathological mechanisms of HT. Lastly, the expression of LTF and MASP-1, two essential glycoproteins, was upregulated in the serum of patients with HT and PTC; the biological functions of LTF and MASP-1 in HT development are worth further studies. These results offer a novel direction for diagnostic and clinical research in the field of HT and PTC.

## Data availability statement

The original contributions presented in the study are publicly available. This data can be found here: ProteomeXchange Consortium [PXD040847].

## Author contributions

YX and XQ performed the majority of the work described in this study and wrote the manuscript. JH, RN, LG, and CX collected the serum samples. YM, JL, and LW performed the data analysis. XQ and LW supervised the project. All authors contributed to the article and approved the submitted version.
