# Supplementary material for: Altered profile of glycosylated proteins in serum samples obtained from patients with Hashimoto′s thyroiditis following depletion of highly abundant proteins
Source: Front Immunol. 2023 Jun 30;14:1182842. doi: 10.3389/fimmu.2023.1182842 (PMC10348014; doi:10.3389/fimmu.2023.1182842)
Supplement: Supplementary file 1 [file DataSheet_1.zip › original figures and tables for identifying the facticity of the study/whole pictures of lectin blot.pptx]

## Slide 1
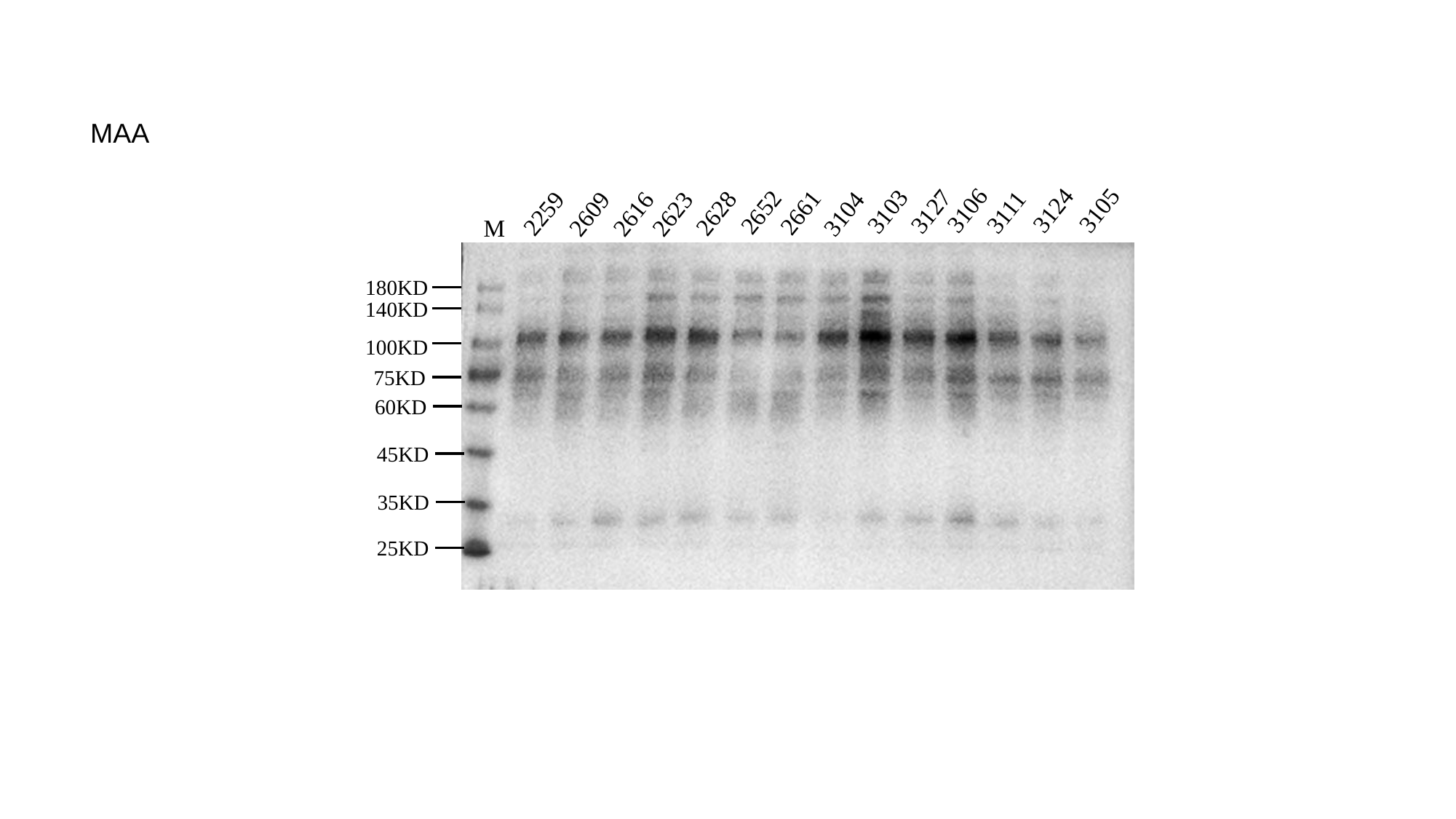

MAA
3105
3106
3124
3127
3111
3103
2652
2661
2628
2259
2616
2609
2623
3104
M
180KD
140KD
100KD
75KD
60KD
45KD
35KD
25KD

## Slide 2
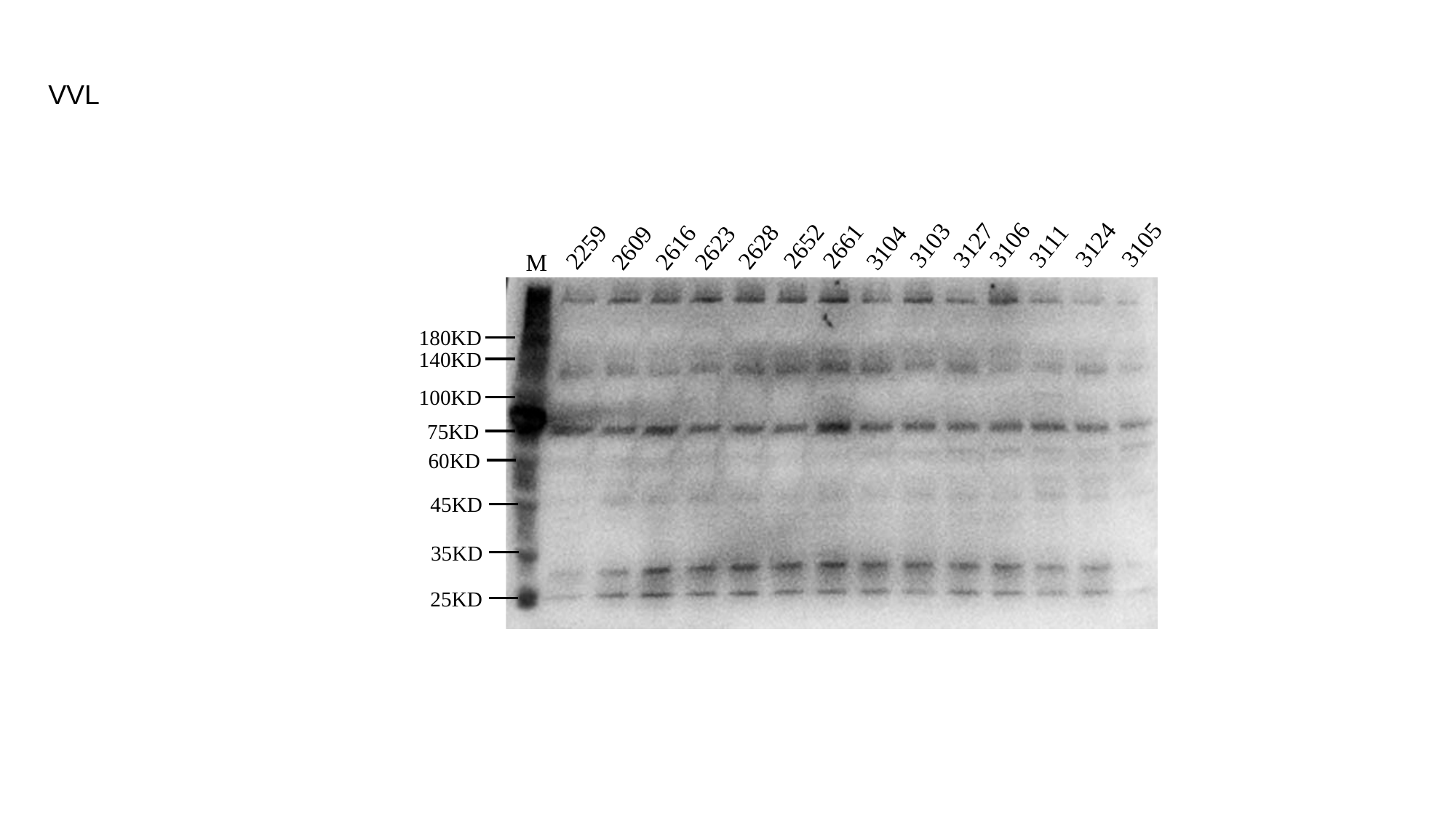

VVL
3105
3106
3124
3127
3111
3103
2652
2661
2628
2259
2616
2609
2623
3104
M
180KD
140KD
100KD
75KD
60KD
45KD
35KD
25KD

## Slide 3
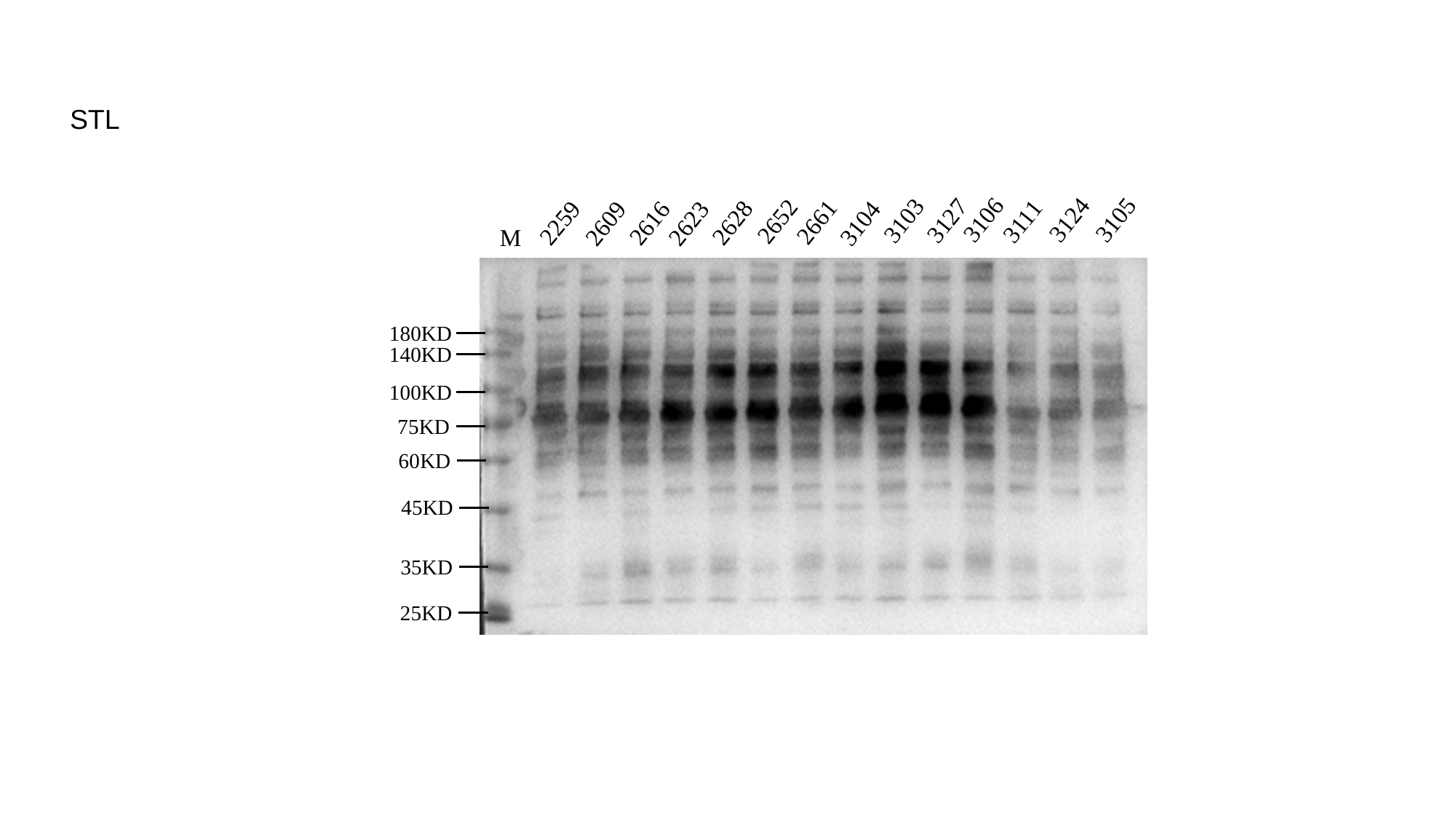

STL
3105
3106
3124
3127
3111
3103
2652
2661
2628
2259
2616
2609
2623
3104
M
180KD
140KD
100KD
75KD
60KD
45KD
35KD
25KD

## Slide 4
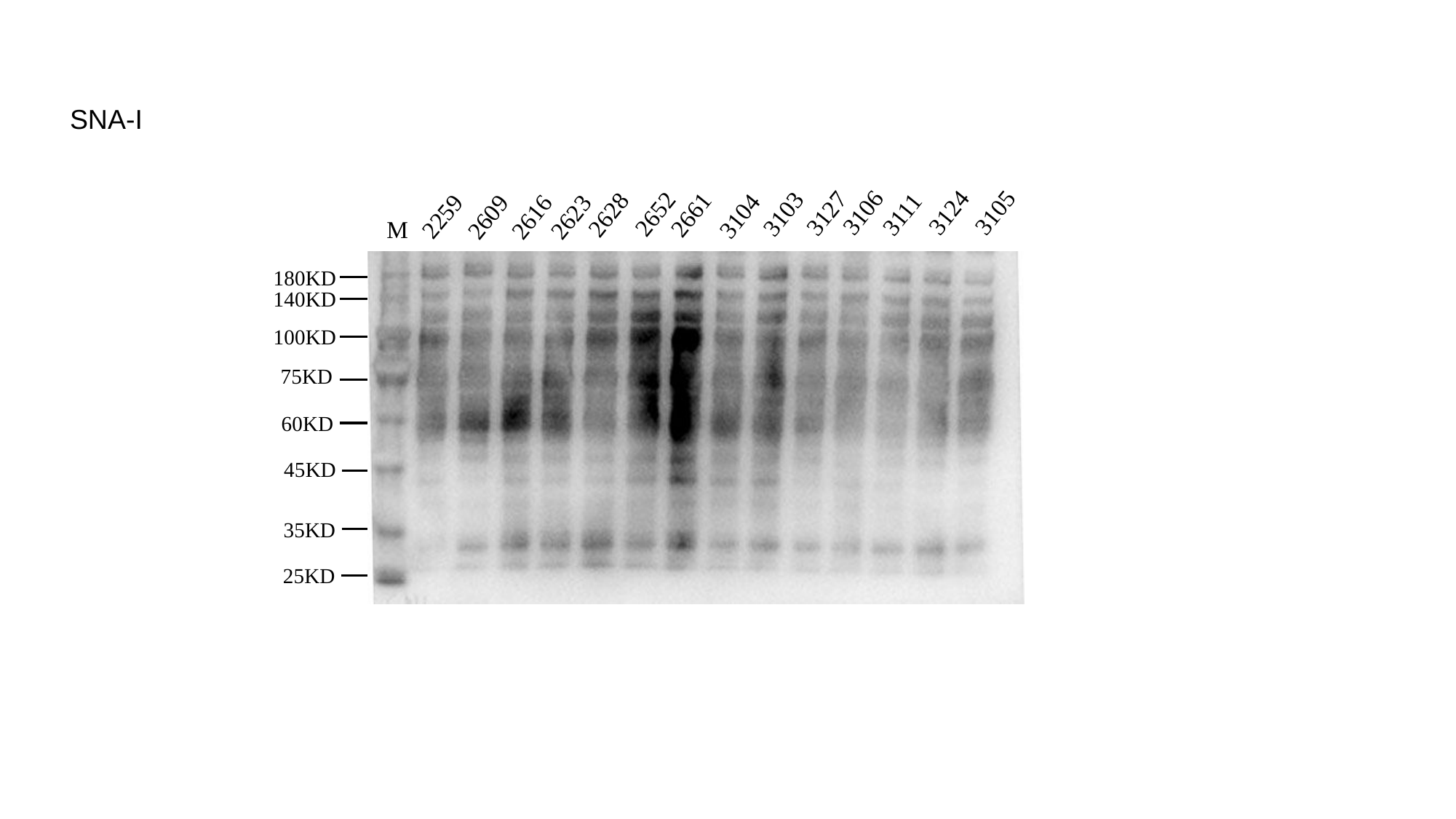

SNA-I
3105
3106
3124
3127
3111
3103
2652
2661
2628
3104
2259
2616
2609
2623
M
180KD
140KD
100KD
75KD
60KD
45KD
35KD
25KD

## Slide 5
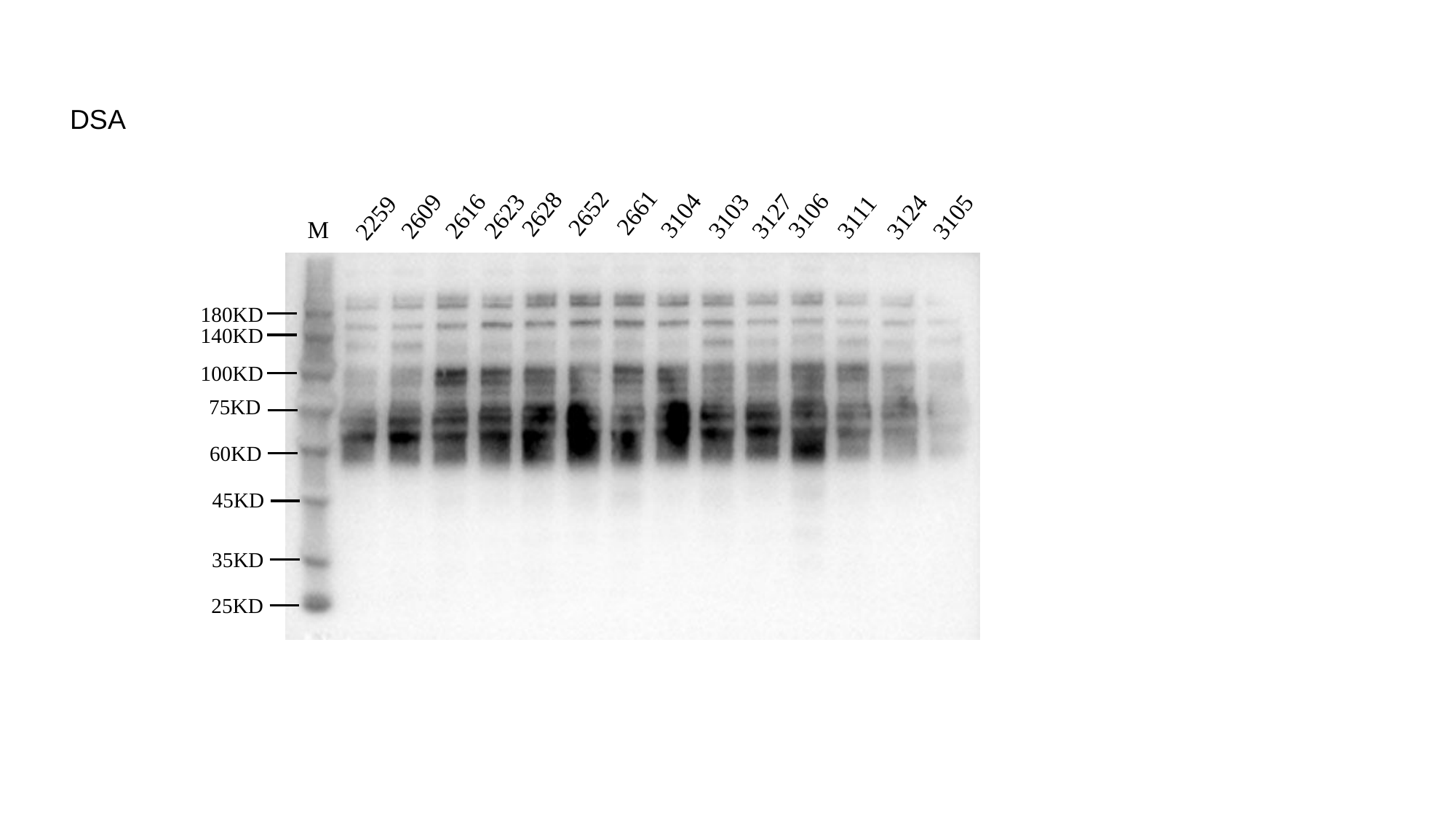

DSA
2661
2652
2628
3106
3104
2616
2609
3111
2623
3127
3103
3105
3124
2259
M
180KD
140KD
100KD
75KD
60KD
45KD
35KD
25KD
