# Supplementary figures and images for: Altered profile of glycosylated proteins in serum samples obtained from patients with Hashimoto′s thyroiditis following depletion of highly abundant proteins
Source: Front Immunol. 2023 Jun 30;14:1182842. doi: 10.3389/fimmu.2023.1182842 (PMC10348014; doi:10.3389/fimmu.2023.1182842)

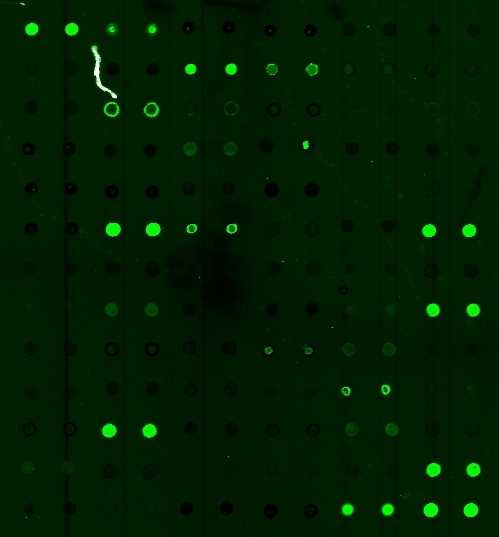

Supplement: Supplementary file 1 [file DataSheet_1.zip › original figures and tables for identifying the facticity of the study/original image for lectin microarray/2259.jpg]

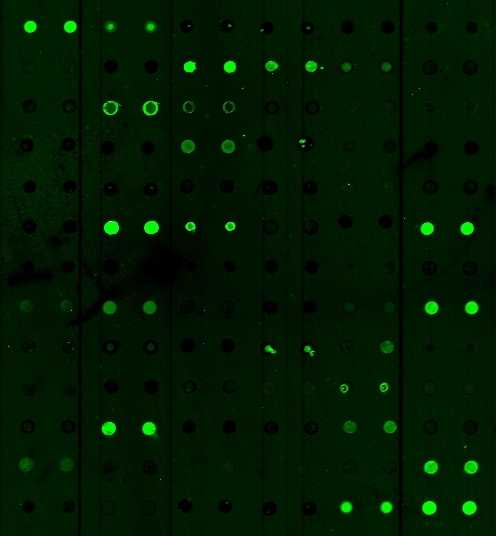

Supplement: Supplementary file 1 [file DataSheet_1.zip › original figures and tables for identifying the facticity of the study/original image for lectin microarray/2339.jpg]

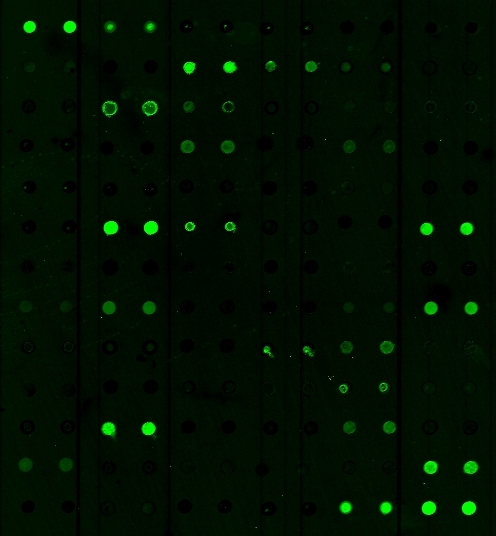

Supplement: Supplementary file 1 [file DataSheet_1.zip › original figures and tables for identifying the facticity of the study/original image for lectin microarray/2480.jpg]

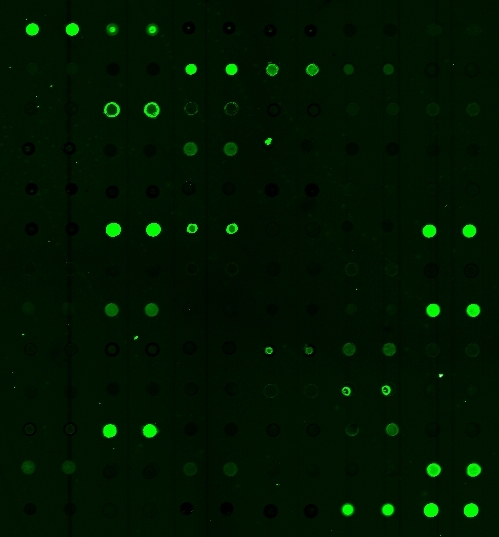

Supplement: Supplementary file 1 [file DataSheet_1.zip › original figures and tables for identifying the facticity of the study/original image for lectin microarray/2569.jpg]

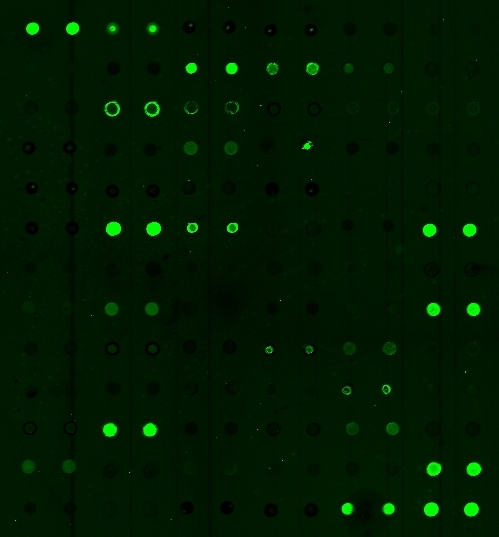

Supplement: Supplementary file 1 [file DataSheet_1.zip › original figures and tables for identifying the facticity of the study/original image for lectin microarray/2602.jpg]

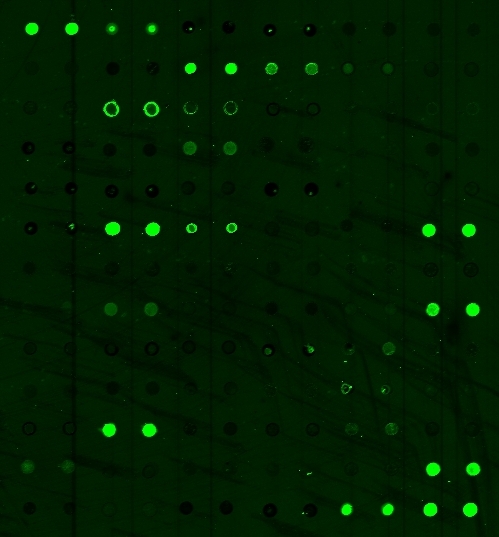

Supplement: Supplementary file 1 [file DataSheet_1.zip › original figures and tables for identifying the facticity of the study/original image for lectin microarray/2609.jpg]

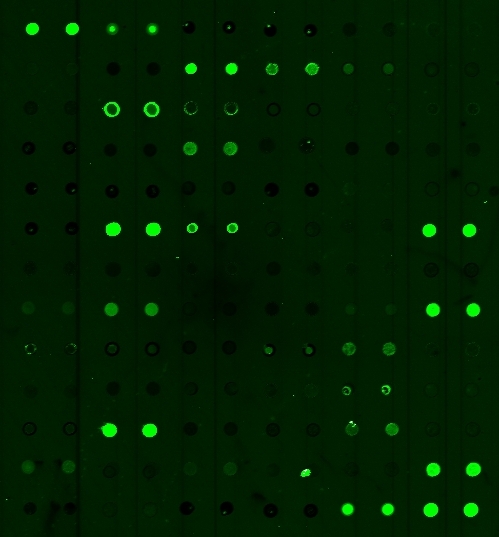

Supplement: Supplementary file 1 [file DataSheet_1.zip › original figures and tables for identifying the facticity of the study/original image for lectin microarray/2610.jpg]

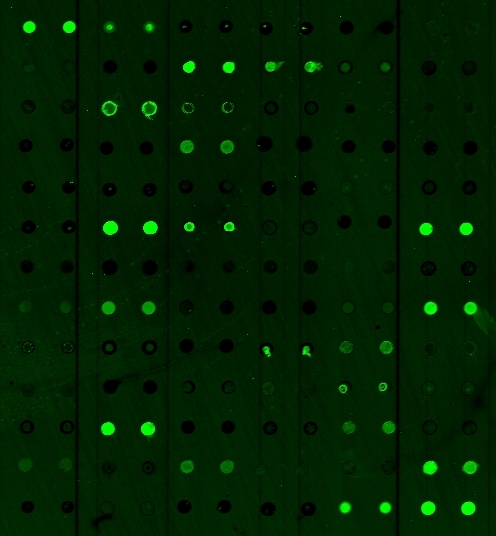

Supplement: Supplementary file 1 [file DataSheet_1.zip › original figures and tables for identifying the facticity of the study/original image for lectin microarray/2611.jpg]

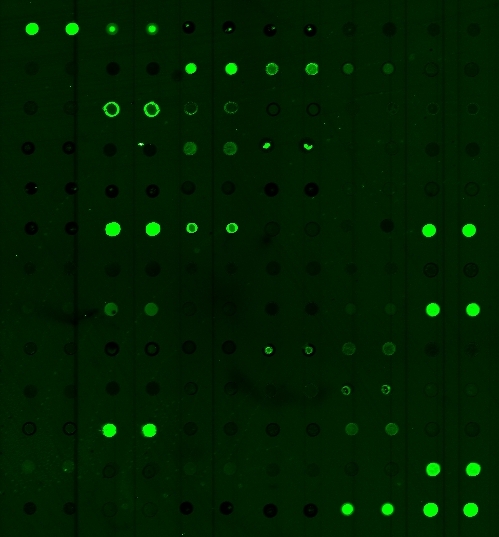

Supplement: Supplementary file 1 [file DataSheet_1.zip › original figures and tables for identifying the facticity of the study/original image for lectin microarray/2612.jpg]

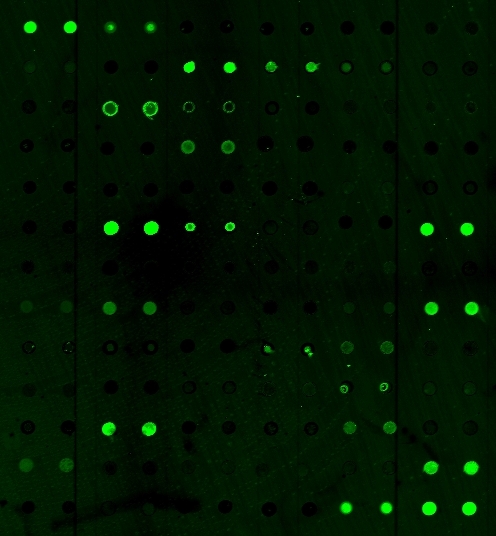

Supplement: Supplementary file 1 [file DataSheet_1.zip › original figures and tables for identifying the facticity of the study/original image for lectin microarray/2620.jpg]

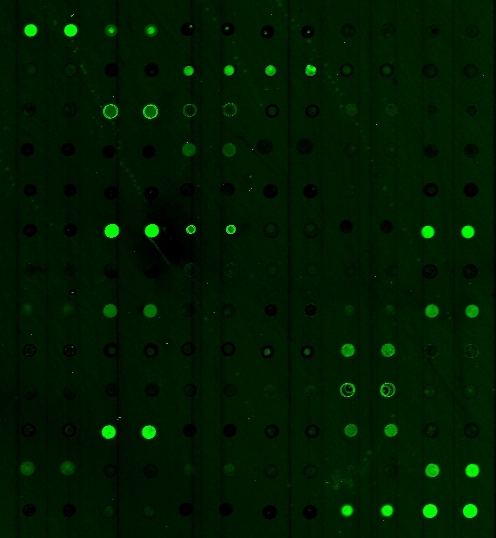

Supplement: Supplementary file 1 [file DataSheet_1.zip › original figures and tables for identifying the facticity of the study/original image for lectin microarray/2623.jpg]

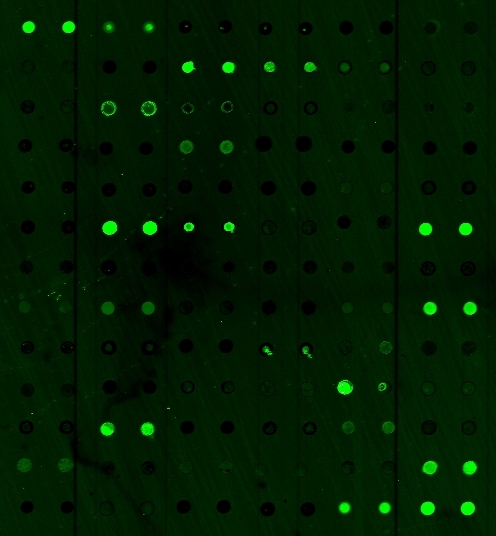

Supplement: Supplementary file 1 [file DataSheet_1.zip › original figures and tables for identifying the facticity of the study/original image for lectin microarray/2626.jpg]

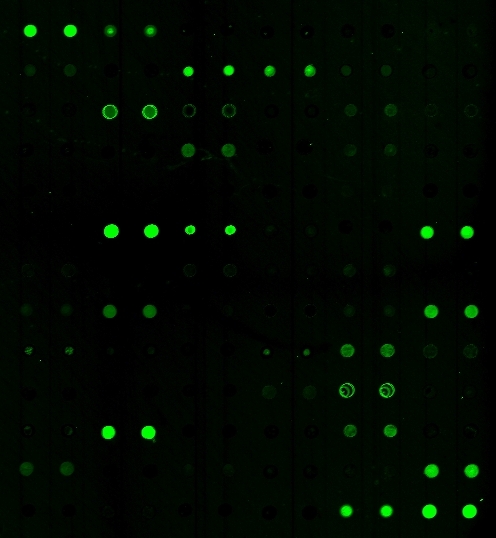

Supplement: Supplementary file 1 [file DataSheet_1.zip › original figures and tables for identifying the facticity of the study/original image for lectin microarray/2628.jpg]

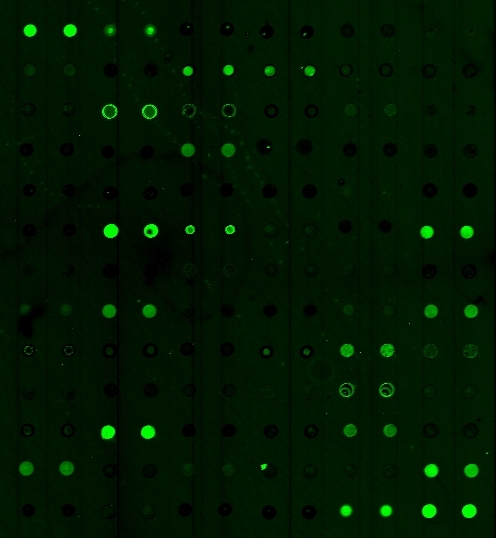

Supplement: Supplementary file 1 [file DataSheet_1.zip › original figures and tables for identifying the facticity of the study/original image for lectin microarray/2629.jpg]

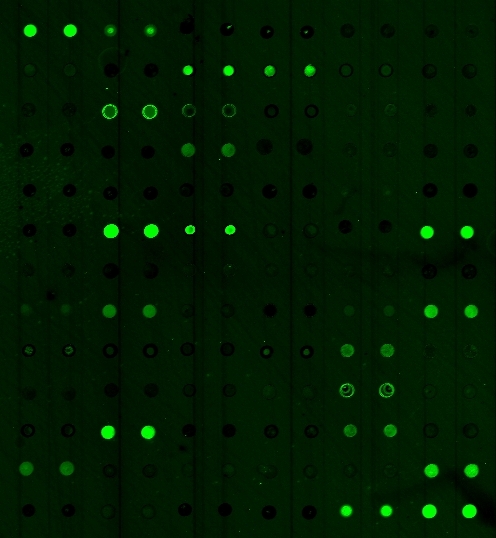

Supplement: Supplementary file 1 [file DataSheet_1.zip › original figures and tables for identifying the facticity of the study/original image for lectin microarray/2630.jpg]

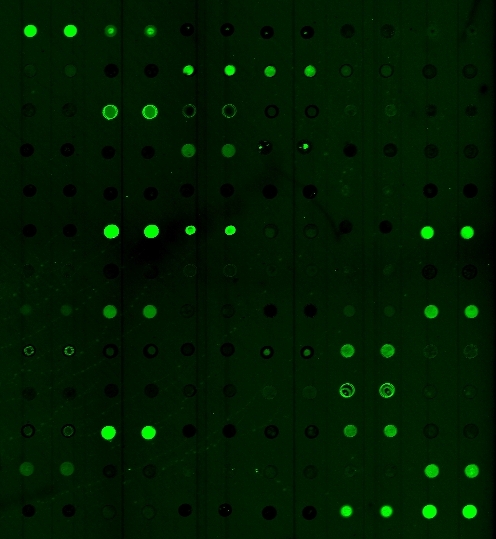

Supplement: Supplementary file 1 [file DataSheet_1.zip › original figures and tables for identifying the facticity of the study/original image for lectin microarray/2631.jpg]

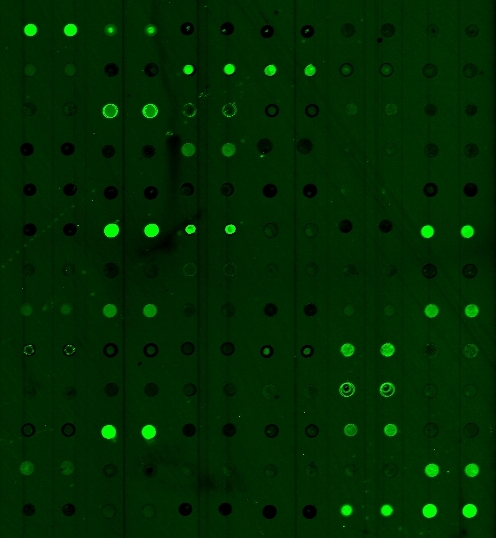

Supplement: Supplementary file 1 [file DataSheet_1.zip › original figures and tables for identifying the facticity of the study/original image for lectin microarray/2635.jpg]

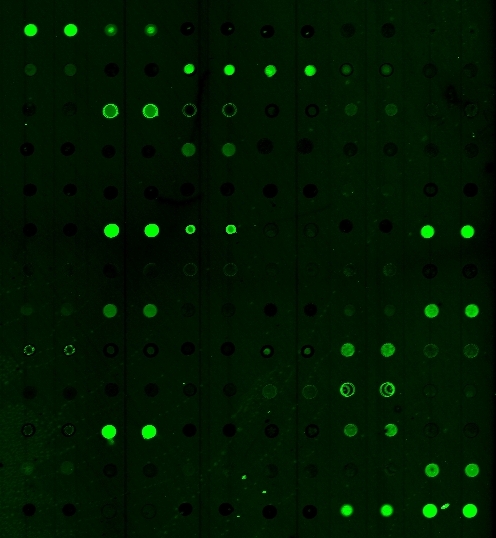

Supplement: Supplementary file 1 [file DataSheet_1.zip › original figures and tables for identifying the facticity of the study/original image for lectin microarray/2637.jpg]

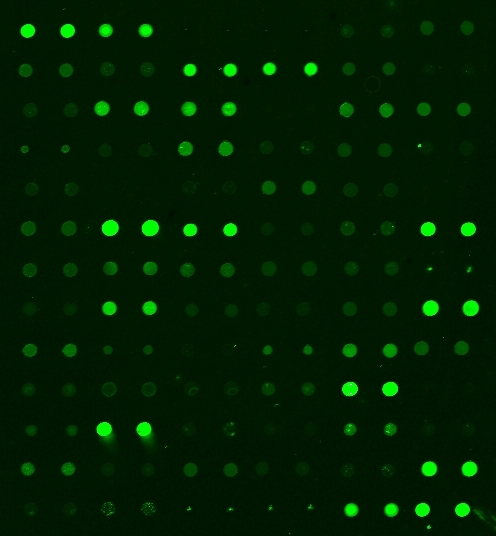

Supplement: Supplementary file 1 [file DataSheet_1.zip › original figures and tables for identifying the facticity of the study/original image for lectin microarray/2639.jpg]

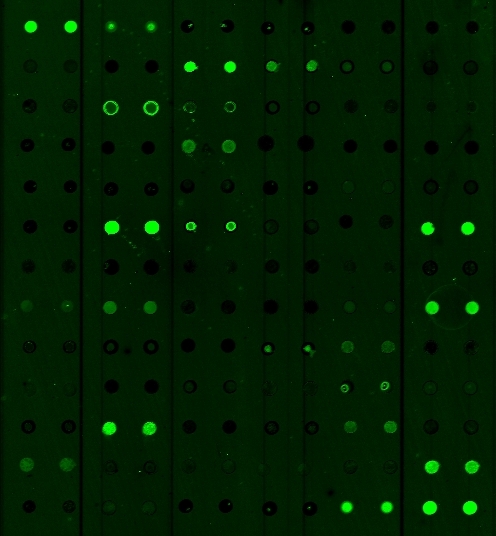

Supplement: Supplementary file 1 [file DataSheet_1.zip › original figures and tables for identifying the facticity of the study/original image for lectin microarray/2642.jpg]

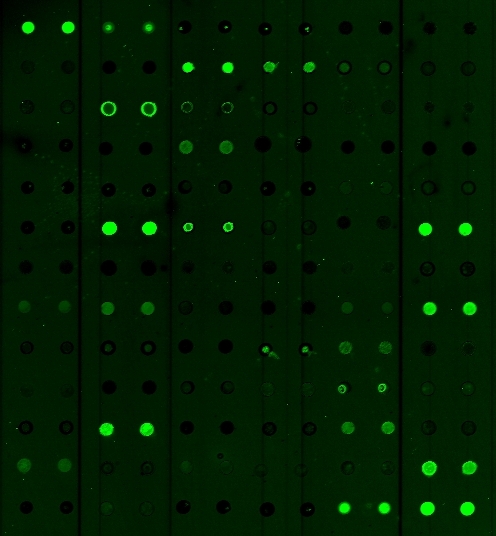

Supplement: Supplementary file 1 [file DataSheet_1.zip › original figures and tables for identifying the facticity of the study/original image for lectin microarray/2644.jpg]

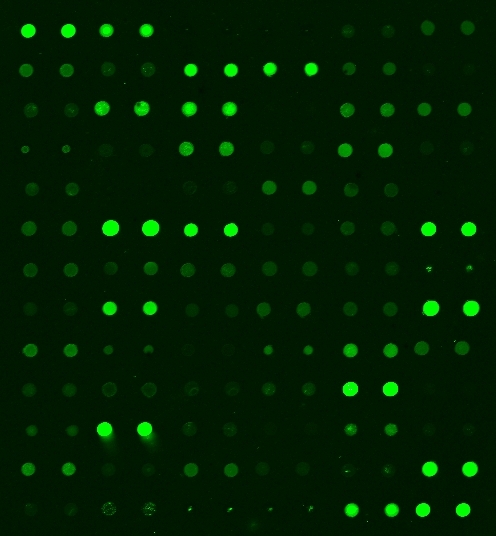

Supplement: Supplementary file 1 [file DataSheet_1.zip › original figures and tables for identifying the facticity of the study/original image for lectin microarray/2647.jpg]

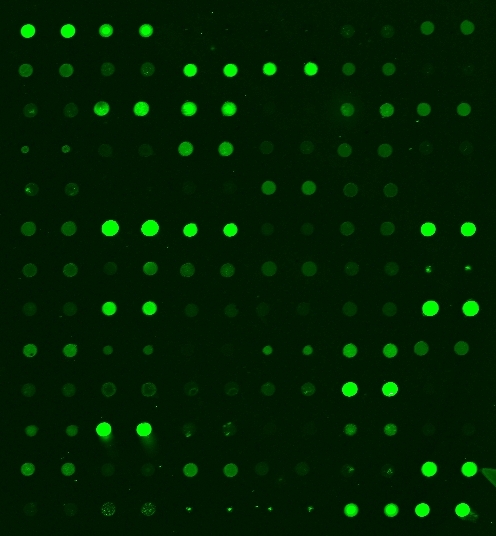

Supplement: Supplementary file 1 [file DataSheet_1.zip › original figures and tables for identifying the facticity of the study/original image for lectin microarray/2649.jpg]

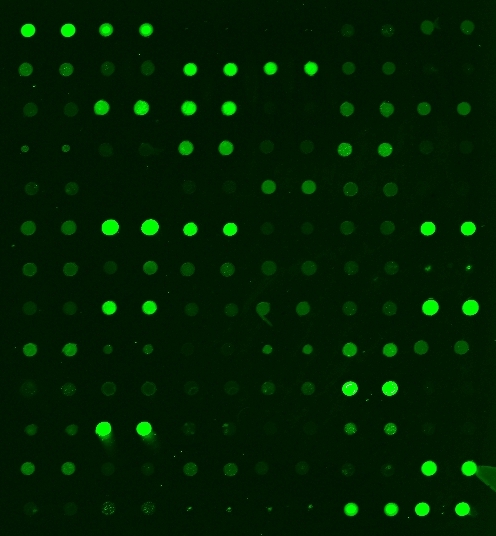

Supplement: Supplementary file 1 [file DataSheet_1.zip › original figures and tables for identifying the facticity of the study/original image for lectin microarray/2652.jpg]

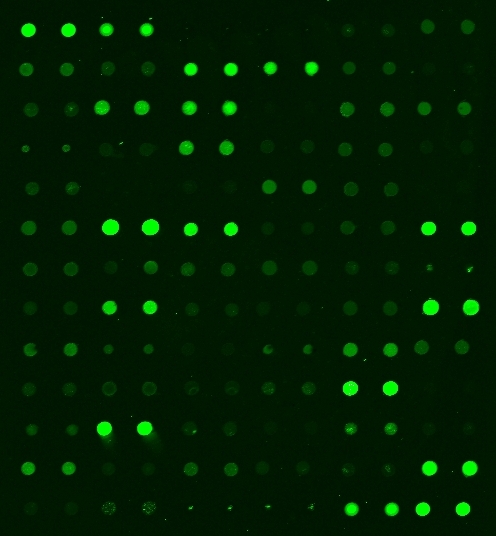

Supplement: Supplementary file 1 [file DataSheet_1.zip › original figures and tables for identifying the facticity of the study/original image for lectin microarray/2656.jpg]

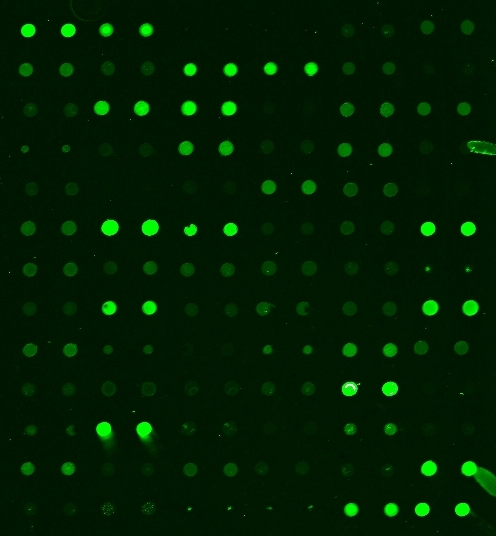

Supplement: Supplementary file 1 [file DataSheet_1.zip › original figures and tables for identifying the facticity of the study/original image for lectin microarray/2661.jpg]

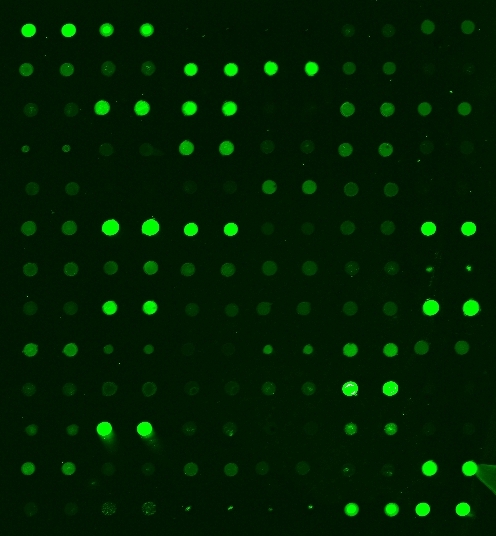

Supplement: Supplementary file 1 [file DataSheet_1.zip › original figures and tables for identifying the facticity of the study/original image for lectin microarray/2662.jpg]

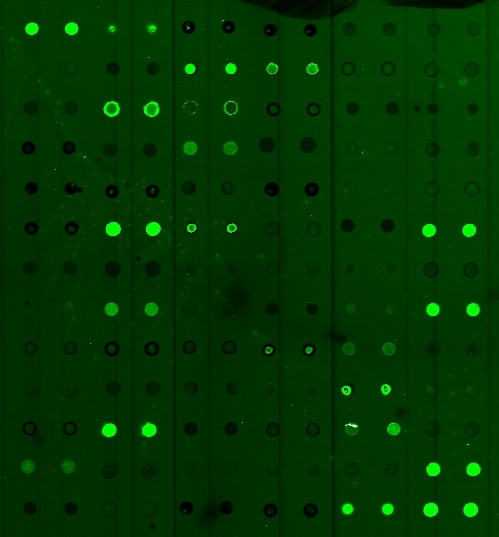

Supplement: Supplementary file 1 [file DataSheet_1.zip › original figures and tables for identifying the facticity of the study/original image for lectin microarray/3100.jpg]

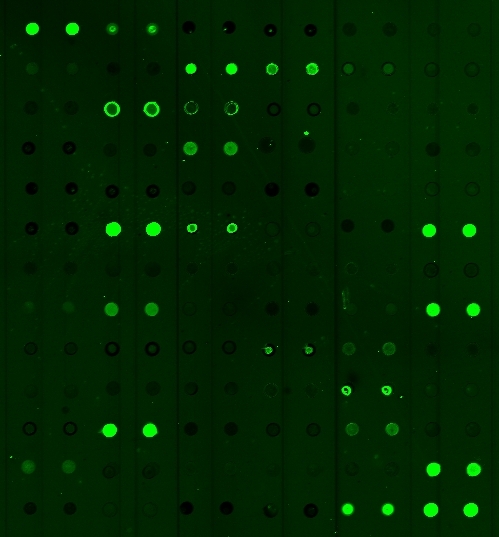

Supplement: Supplementary file 1 [file DataSheet_1.zip › original figures and tables for identifying the facticity of the study/original image for lectin microarray/3101.jpg]

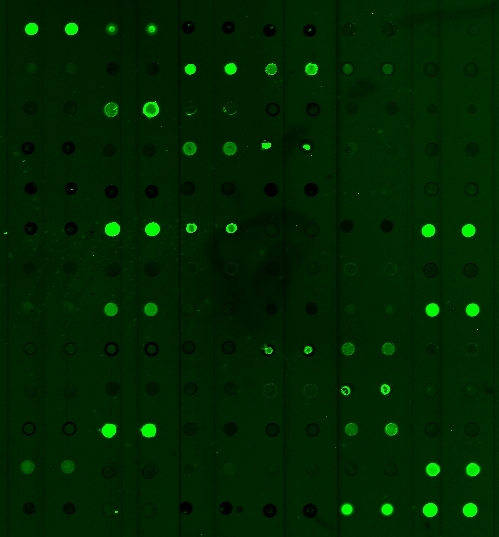

Supplement: Supplementary file 1 [file DataSheet_1.zip › original figures and tables for identifying the facticity of the study/original image for lectin microarray/3102.jpg]

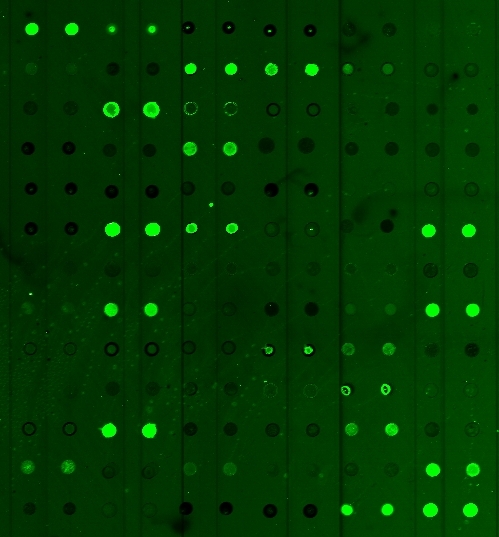

Supplement: Supplementary file 1 [file DataSheet_1.zip › original figures and tables for identifying the facticity of the study/original image for lectin microarray/3103.jpg]

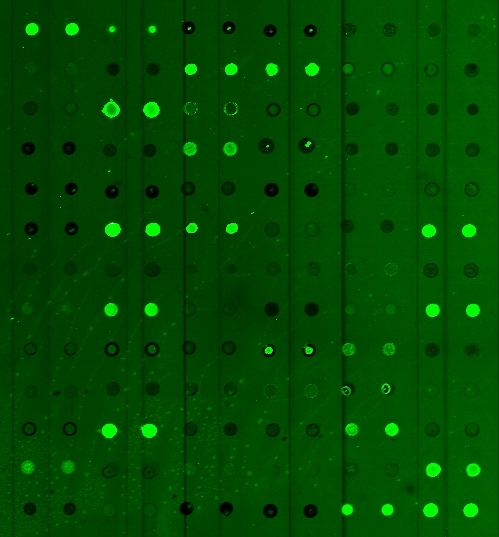

Supplement: Supplementary file 1 [file DataSheet_1.zip › original figures and tables for identifying the facticity of the study/original image for lectin microarray/3104.jpg]

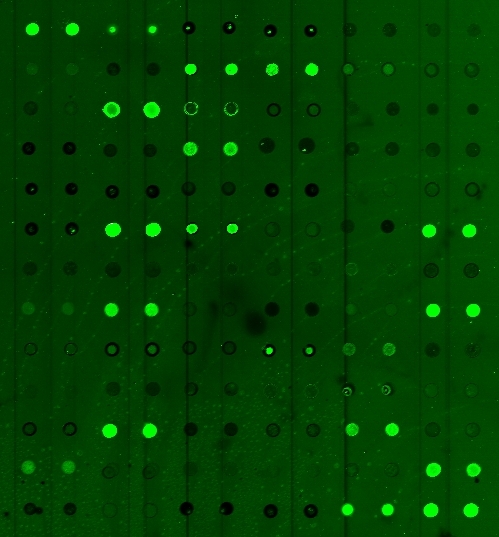

Supplement: Supplementary file 1 [file DataSheet_1.zip › original figures and tables for identifying the facticity of the study/original image for lectin microarray/3105.jpg]

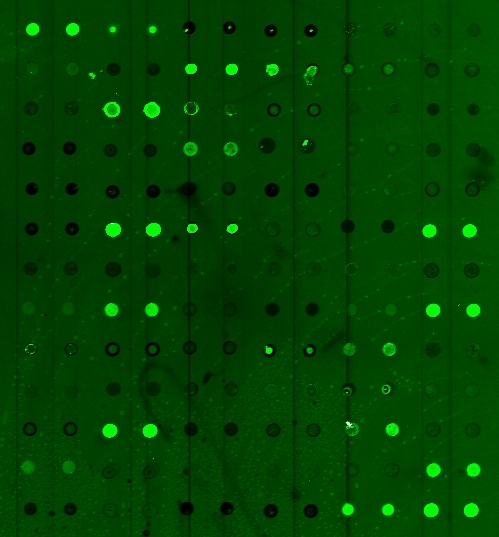

Supplement: Supplementary file 1 [file DataSheet_1.zip › original figures and tables for identifying the facticity of the study/original image for lectin microarray/3106.jpg]

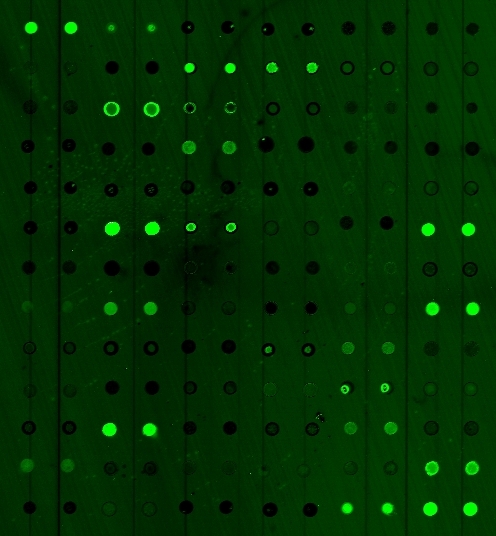

Supplement: Supplementary file 1 [file DataSheet_1.zip › original figures and tables for identifying the facticity of the study/original image for lectin microarray/3107.jpg]

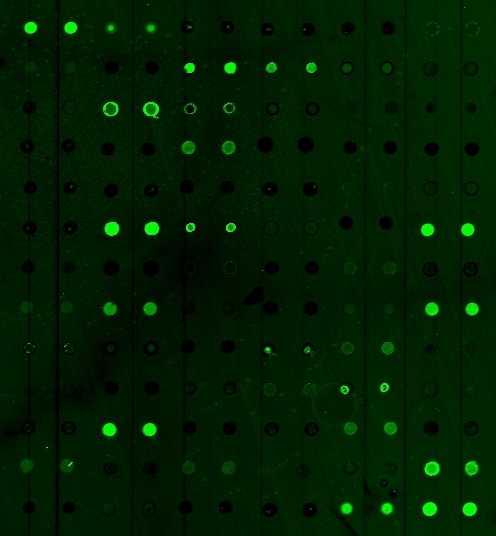

Supplement: Supplementary file 1 [file DataSheet_1.zip › original figures and tables for identifying the facticity of the study/original image for lectin microarray/3108.jpg]

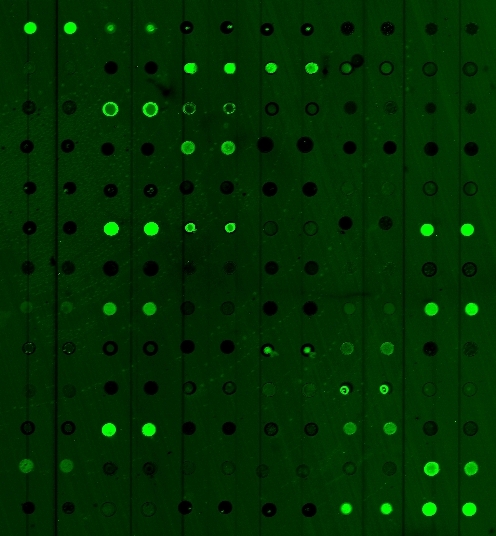

Supplement: Supplementary file 1 [file DataSheet_1.zip › original figures and tables for identifying the facticity of the study/original image for lectin microarray/3109.jpg]

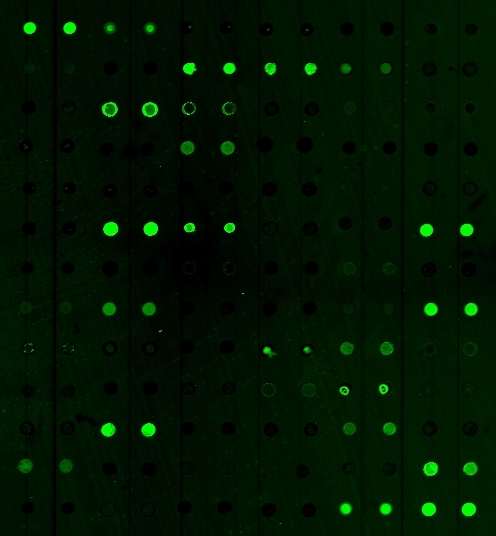

Supplement: Supplementary file 1 [file DataSheet_1.zip › original figures and tables for identifying the facticity of the study/original image for lectin microarray/3110.jpg]

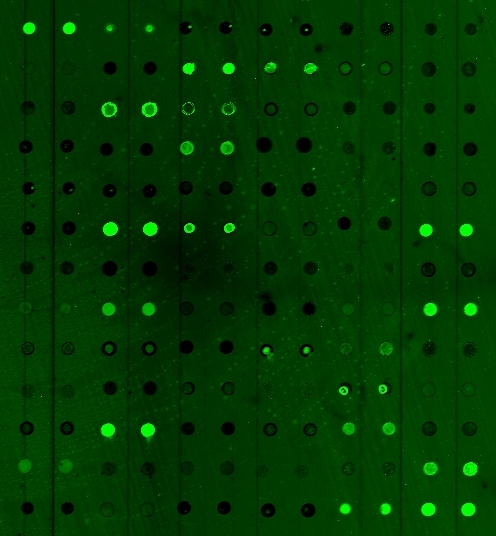

Supplement: Supplementary file 1 [file DataSheet_1.zip › original figures and tables for identifying the facticity of the study/original image for lectin microarray/3111.jpg]

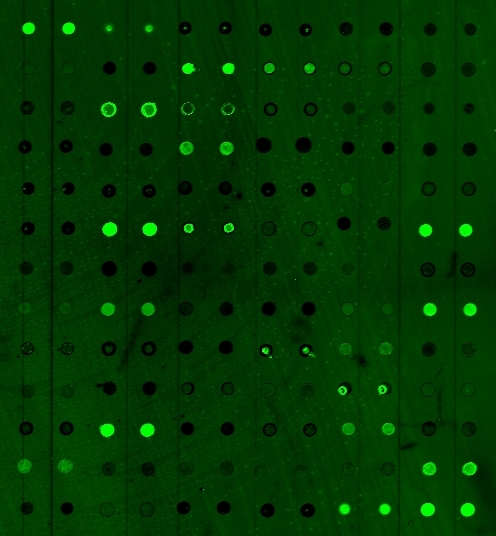

Supplement: Supplementary file 1 [file DataSheet_1.zip › original figures and tables for identifying the facticity of the study/original image for lectin microarray/3112.jpg]

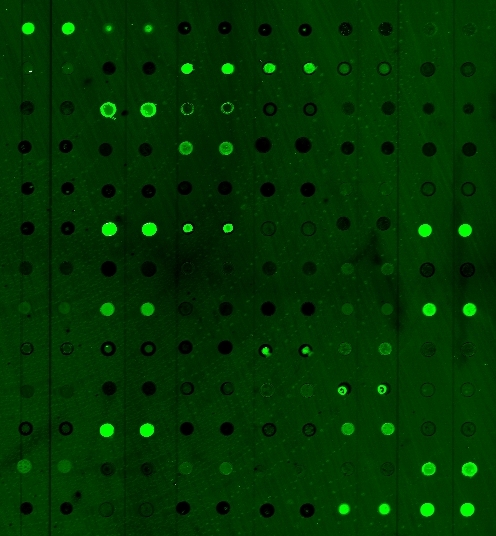

Supplement: Supplementary file 1 [file DataSheet_1.zip › original figures and tables for identifying the facticity of the study/original image for lectin microarray/3113.jpg]

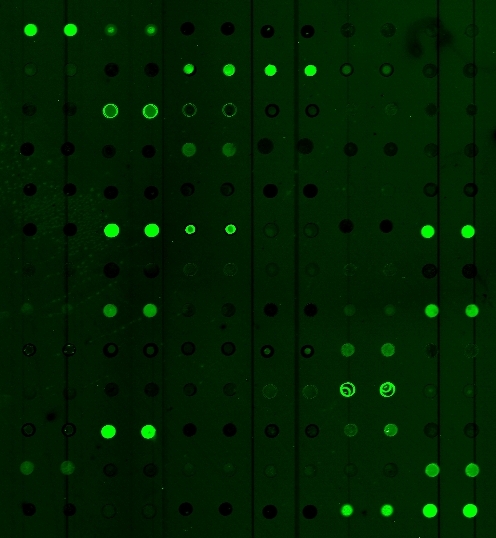

Supplement: Supplementary file 1 [file DataSheet_1.zip › original figures and tables for identifying the facticity of the study/original image for lectin microarray/3114.jpg]

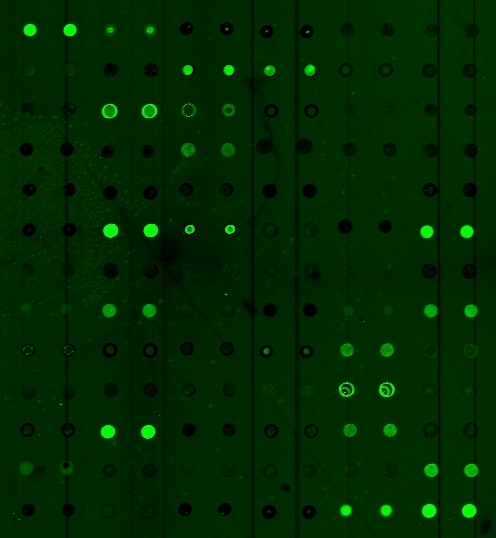

Supplement: Supplementary file 1 [file DataSheet_1.zip › original figures and tables for identifying the facticity of the study/original image for lectin microarray/3115.jpg]

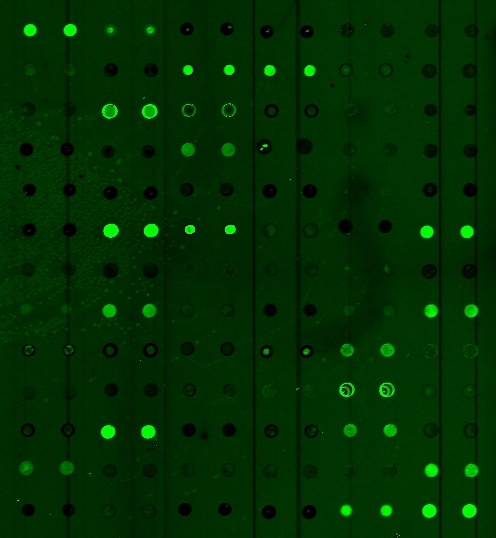

Supplement: Supplementary file 1 [file DataSheet_1.zip › original figures and tables for identifying the facticity of the study/original image for lectin microarray/3116.jpg]

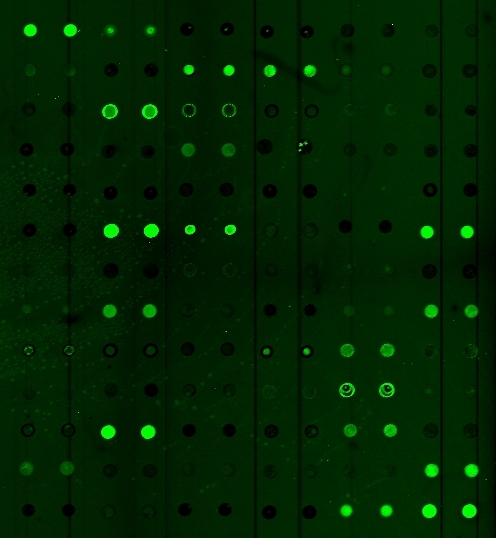

Supplement: Supplementary file 1 [file DataSheet_1.zip › original figures and tables for identifying the facticity of the study/original image for lectin microarray/3117.jpg]

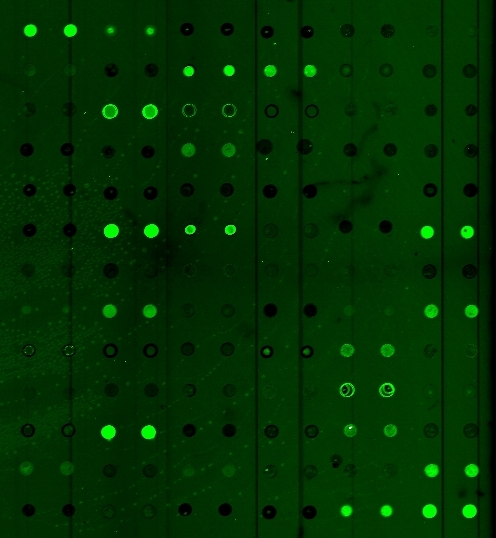

Supplement: Supplementary file 1 [file DataSheet_1.zip › original figures and tables for identifying the facticity of the study/original image for lectin microarray/3118.jpg]

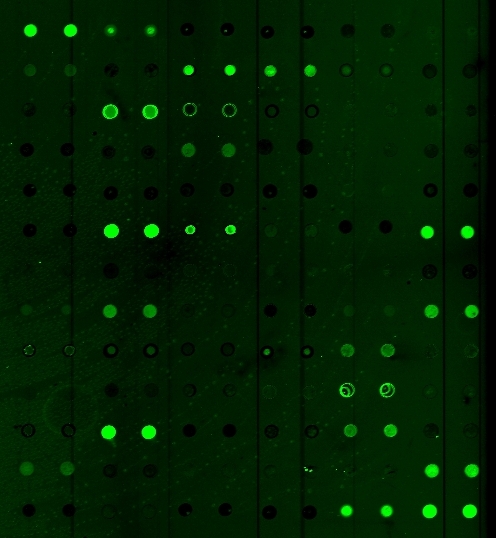

Supplement: Supplementary file 1 [file DataSheet_1.zip › original figures and tables for identifying the facticity of the study/original image for lectin microarray/3119.jpg]

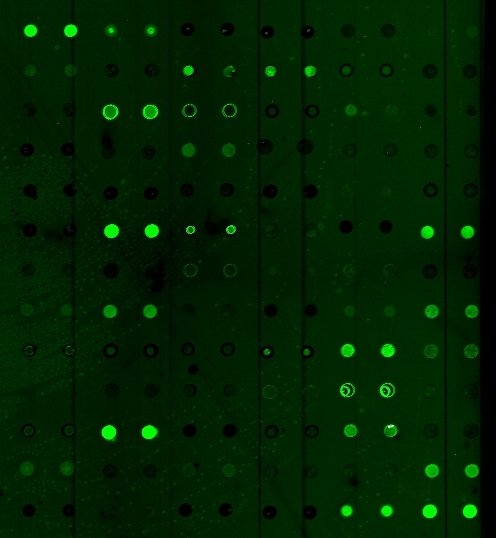

Supplement: Supplementary file 1 [file DataSheet_1.zip › original figures and tables for identifying the facticity of the study/original image for lectin microarray/3120.jpg]

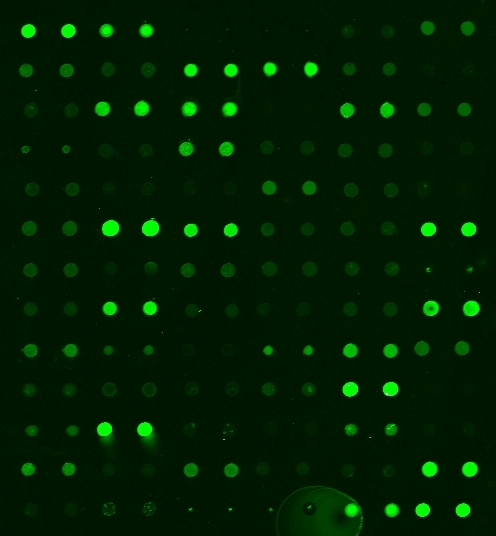

Supplement: Supplementary file 1 [file DataSheet_1.zip › original figures and tables for identifying the facticity of the study/original image for lectin microarray/3122.jpg]

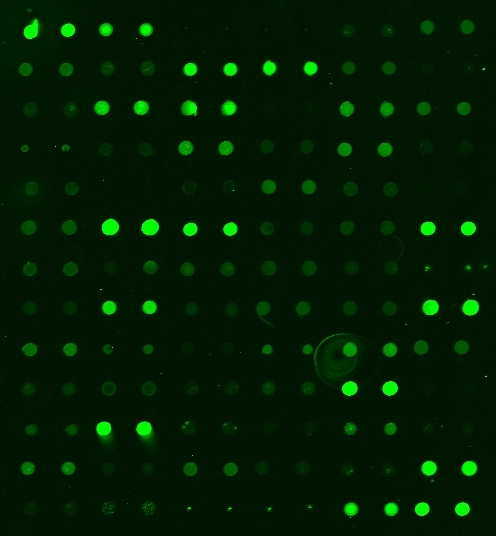

Supplement: Supplementary file 1 [file DataSheet_1.zip › original figures and tables for identifying the facticity of the study/original image for lectin microarray/3123.jpg]

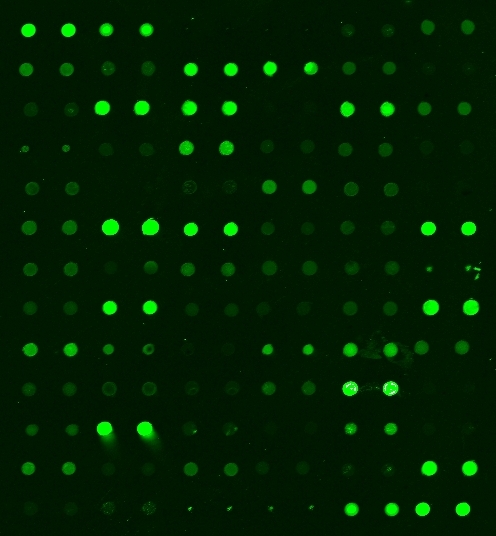

Supplement: Supplementary file 1 [file DataSheet_1.zip › original figures and tables for identifying the facticity of the study/original image for lectin microarray/3124.jpg]

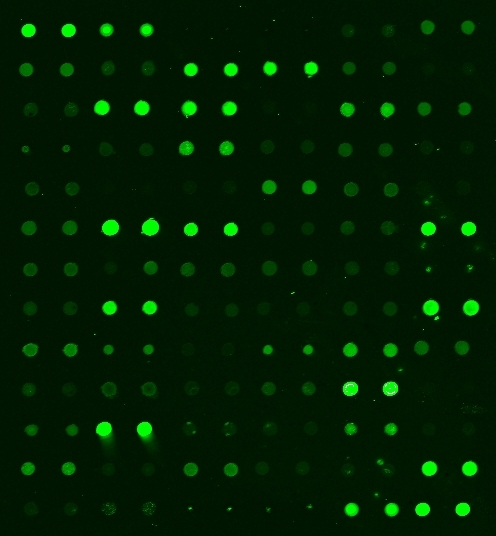

Supplement: Supplementary file 1 [file DataSheet_1.zip › original figures and tables for identifying the facticity of the study/original image for lectin microarray/3126.jpg]

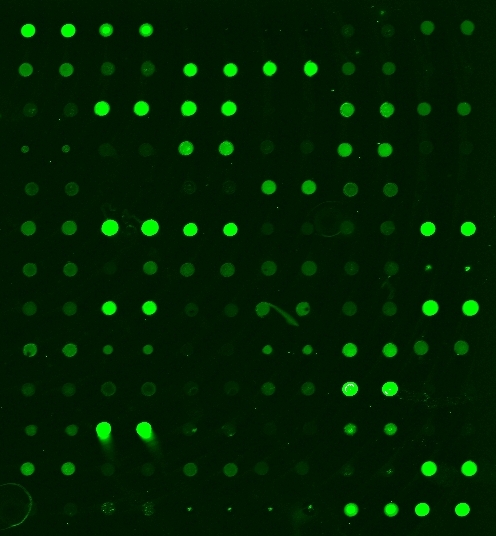

Supplement: Supplementary file 1 [file DataSheet_1.zip › original figures and tables for identifying the facticity of the study/original image for lectin microarray/3127.jpg]

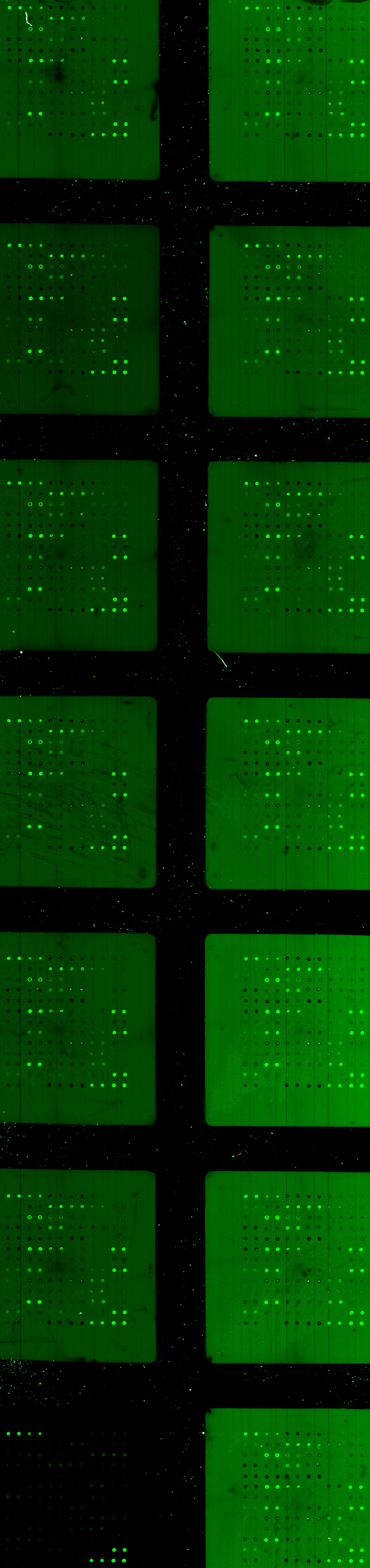

Supplement: Supplementary file 1 [file DataSheet_1.zip › original figures and tables for identifying the facticity of the study/original image for lectin microarray/lectin microarray 1.jpg]

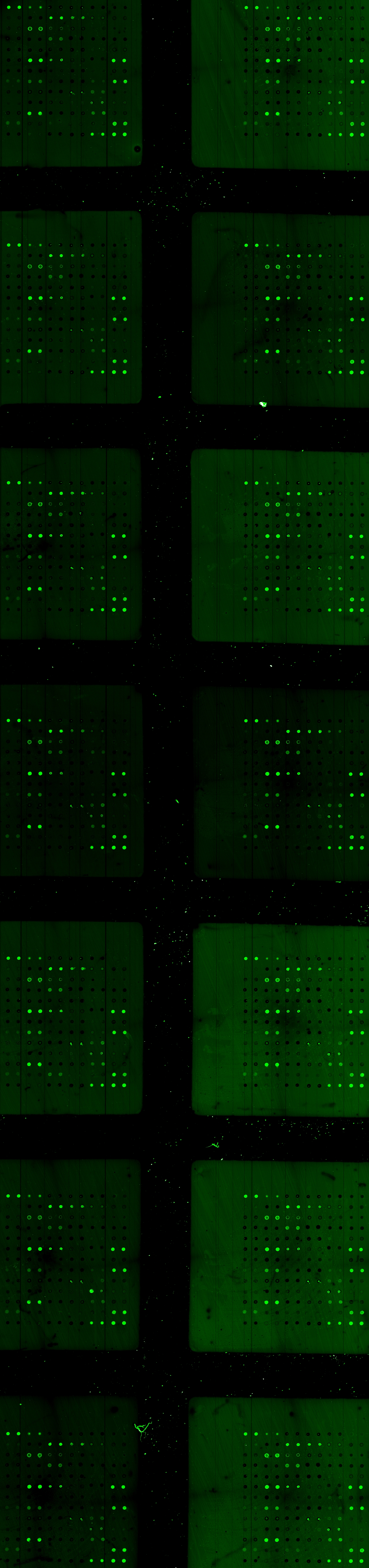

Supplement: Supplementary file 1 [file DataSheet_1.zip › original figures and tables for identifying the facticity of the study/original image for lectin microarray/lectin microarray 2.jpg]

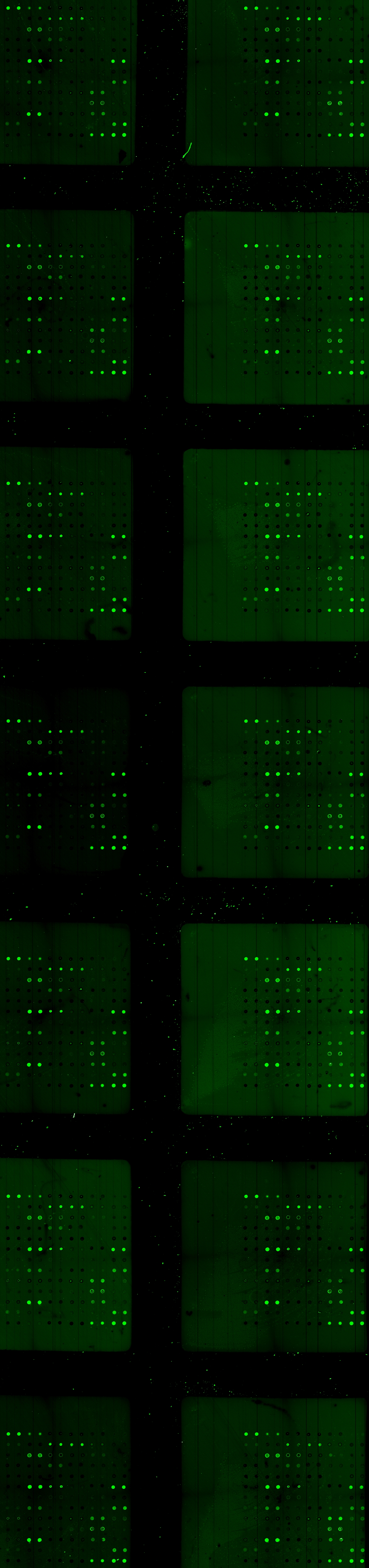

Supplement: Supplementary file 1 [file DataSheet_1.zip › original figures and tables for identifying the facticity of the study/original image for lectin microarray/lectin microarray 3.jpg]

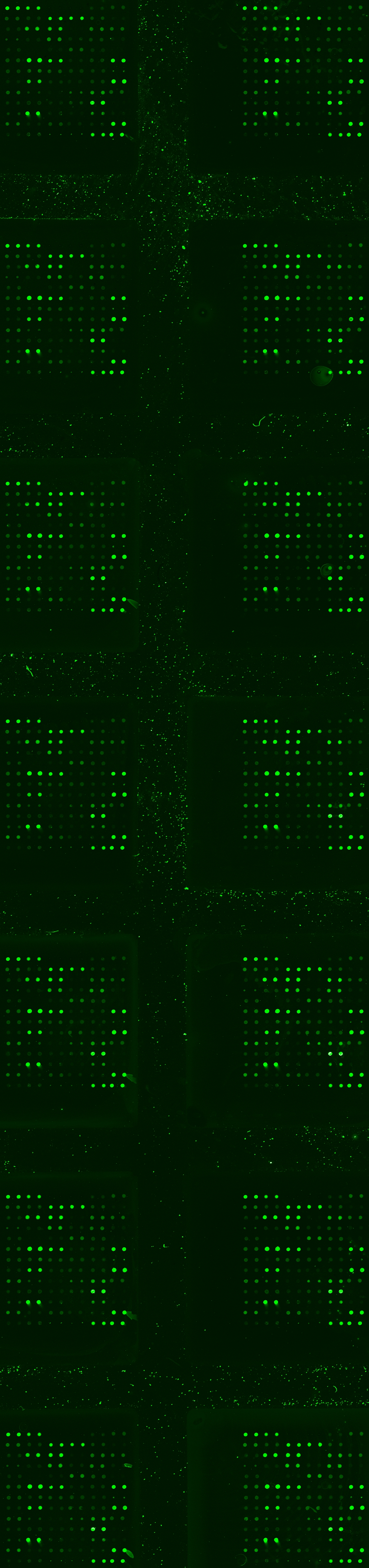

Supplement: Supplementary file 1 [file DataSheet_1.zip › original figures and tables for identifying the facticity of the study/original image for lectin microarray/lectin microarray 4.jpg]
