# Supplementary material for: Altered profile of glycosylated proteins in serum samples obtained from patients with Hashimoto′s thyroiditis following depletion of highly abundant proteins
Source: Front Immunol. 2023 Jun 30;14:1182842. doi: 10.3389/fimmu.2023.1182842 (PMC10348014; doi:10.3389/fimmu.2023.1182842)
Supplement: Supplementary file 3 [file Presentation_1.pptx]

## Slide 1
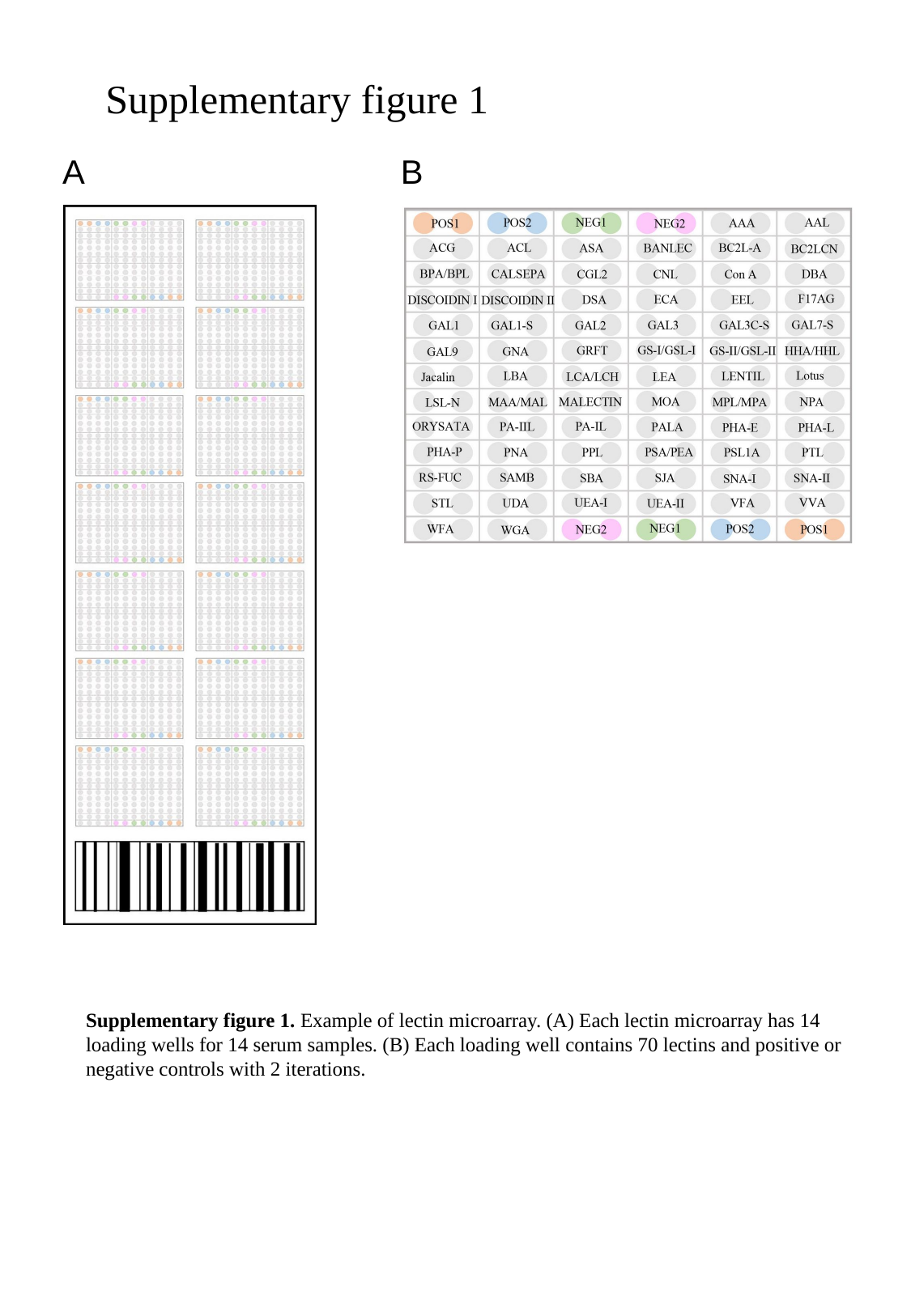

Supplementary figure 1
A
B
Supplementary figure 1. Example of lectin microarray. (A) Each lectin microarray has 14 loading wells for 14 serum samples. (B) Each loading well contains 70 lectins and positive or negative controls with 2 iterations.

## Slide 2
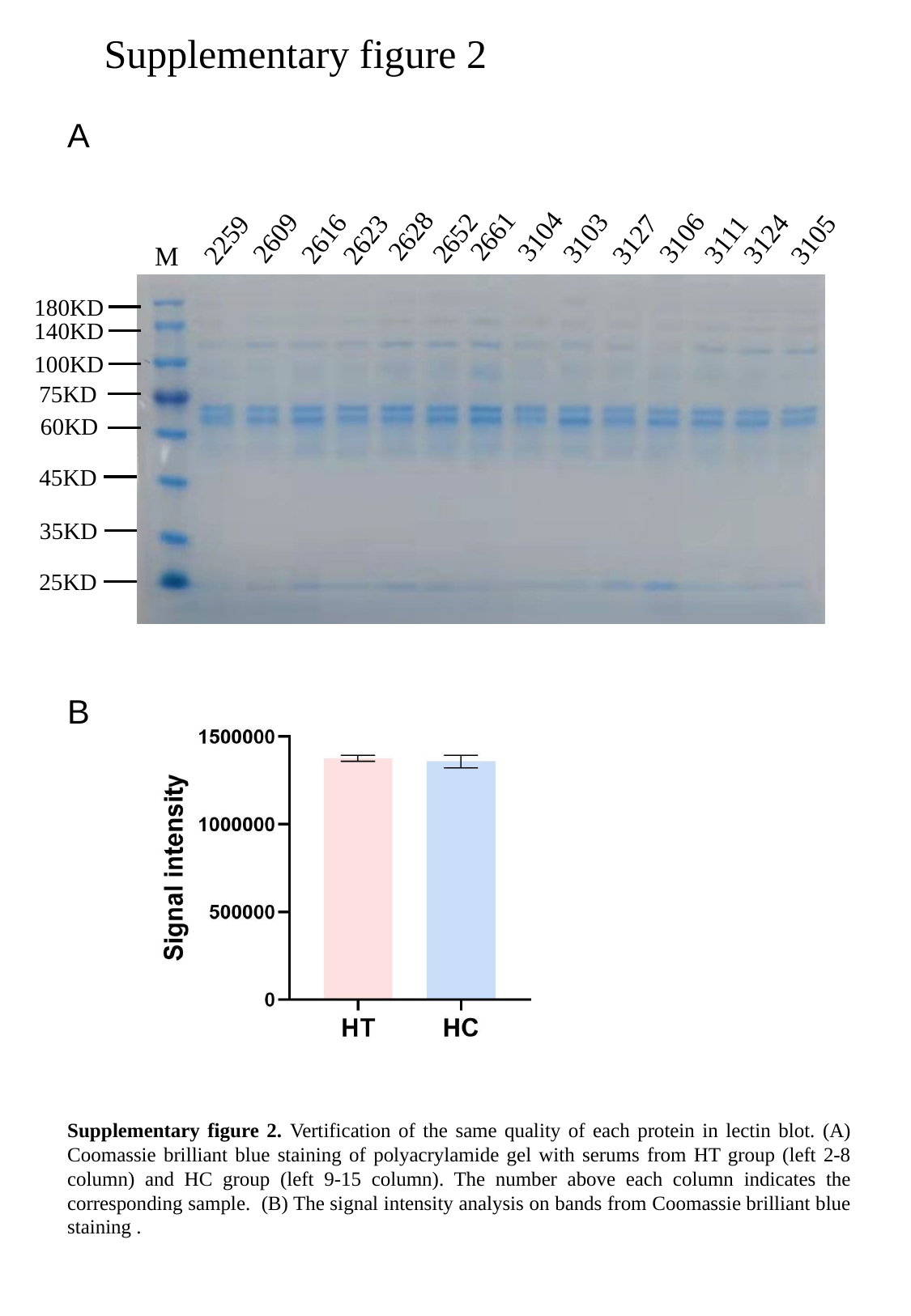

Supplementary figure 2
A
2628
2661
3104
2609
3103
2652
3106
3111
3124
2616
2623
3127
3105
2259
M
180KD
140KD
100KD
75KD
60KD
45KD
35KD
25KD
B
Supplementary figure 2. Vertification of the same quality of each protein in lectin blot. (A) Coomassie brilliant blue staining of polyacrylamide gel with serums from HT group (left 2-8 column) and HC group (left 9-15 column). The number above each column indicates the corresponding sample. (B) The signal intensity analysis on bands from Coomassie brilliant blue staining .

## Slide 3
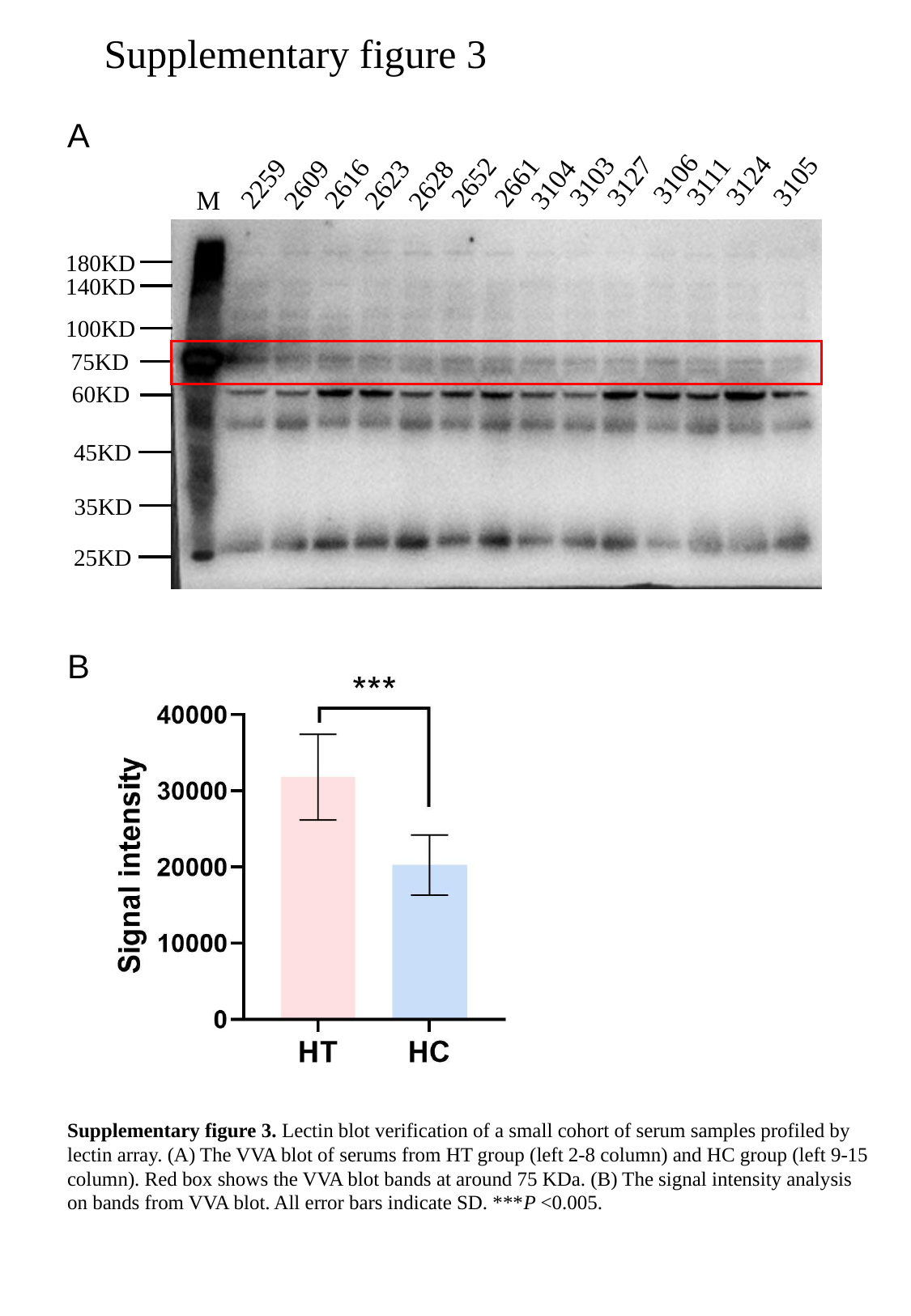

Supplementary figure 3
A
3106
3111
3124
3103
3127
3105
2652
2661
2259
2616
2609
2623
3104
2628
M
180KD
140KD
100KD
75KD
60KD
45KD
35KD
25KD
B
Supplementary figure 3. Lectin blot verification of a small cohort of serum samples profiled by lectin array. (A) The VVA blot of serums from HT group (left 2-8 column) and HC group (left 9-15 column). Red box shows the VVA blot bands at around 75 KDa. (B) The signal intensity analysis on bands from VVA blot. All error bars indicate SD. ***P <0.005.

## Slide 4
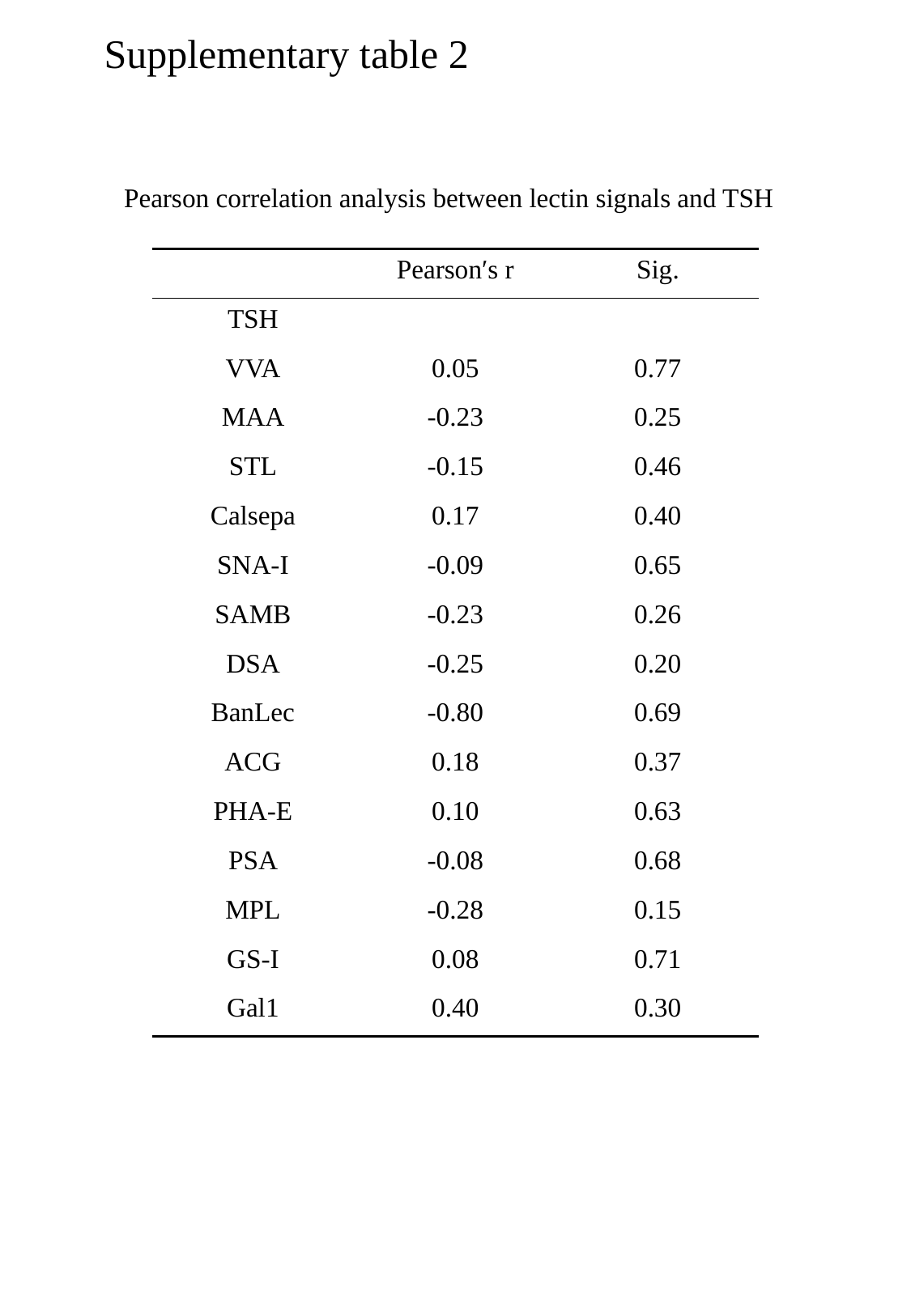

Supplementary table 2
Pearson correlation analysis between lectin signals and TSH
| | Pearson′s r | Sig. |
| --- | --- | --- |
| TSH | | |
| VVA | 0.05 | 0.77 |
| MAA | -0.23 | 0.25 |
| STL | -0.15 | 0.46 |
| Calsepa | 0.17 | 0.40 |
| SNA-I | -0.09 | 0.65 |
| SAMB | -0.23 | 0.26 |
| DSA | -0.25 | 0.20 |
| BanLec | -0.80 | 0.69 |
| ACG | 0.18 | 0.37 |
| PHA-E | 0.10 | 0.63 |
| PSA | -0.08 | 0.68 |
| MPL | -0.28 | 0.15 |
| GS-I | 0.08 | 0.71 |
| Gal1 | 0.40 | 0.30 |

## Slide 5
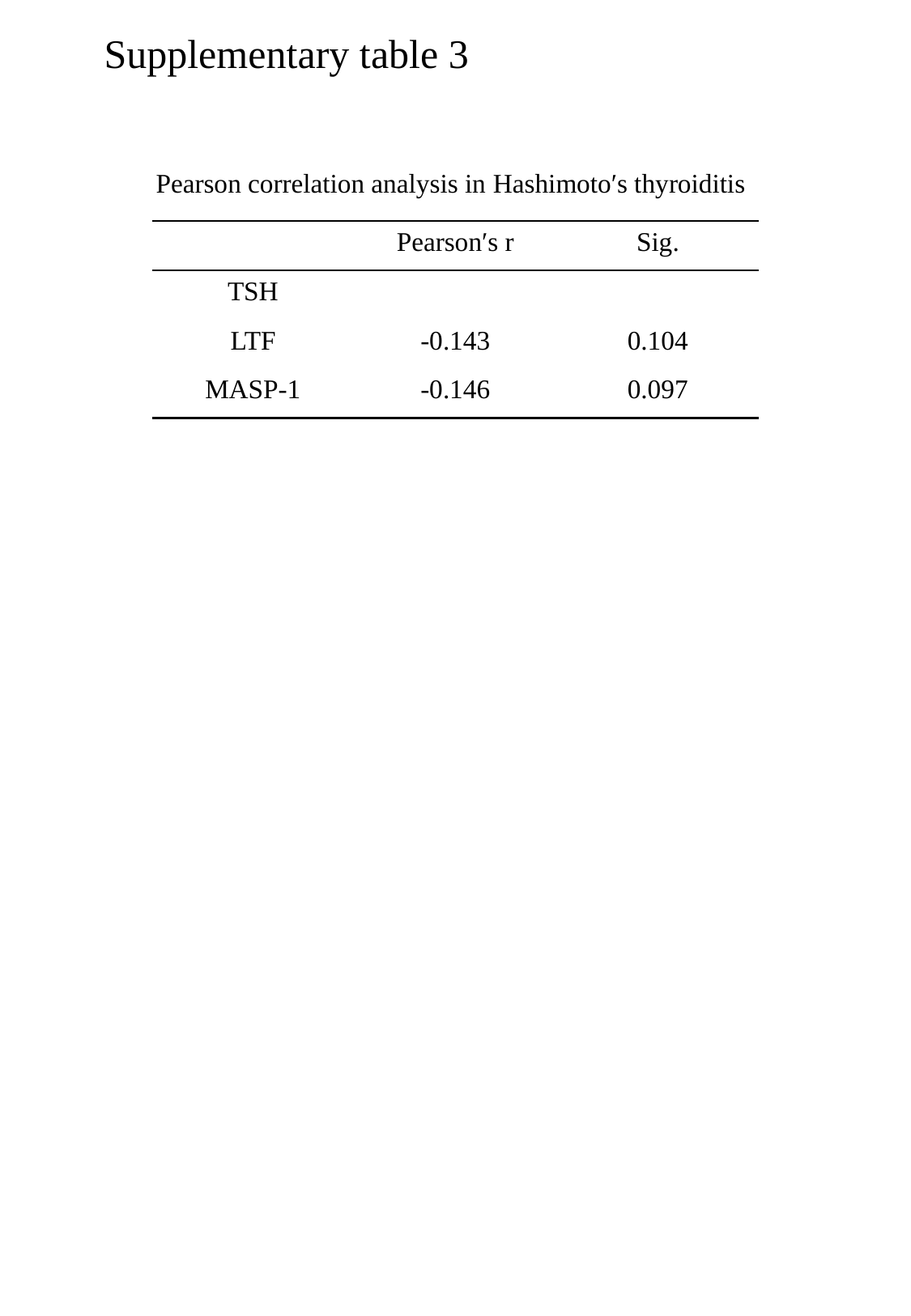

Supplementary table 3
Pearson correlation analysis in Hashimoto′s thyroiditis
| | Pearson′s r | Sig. |
| --- | --- | --- |
| TSH | | |
| LTF | -0.143 | 0.104 |
| MASP-1 | -0.146 | 0.097 |

## Slide 6
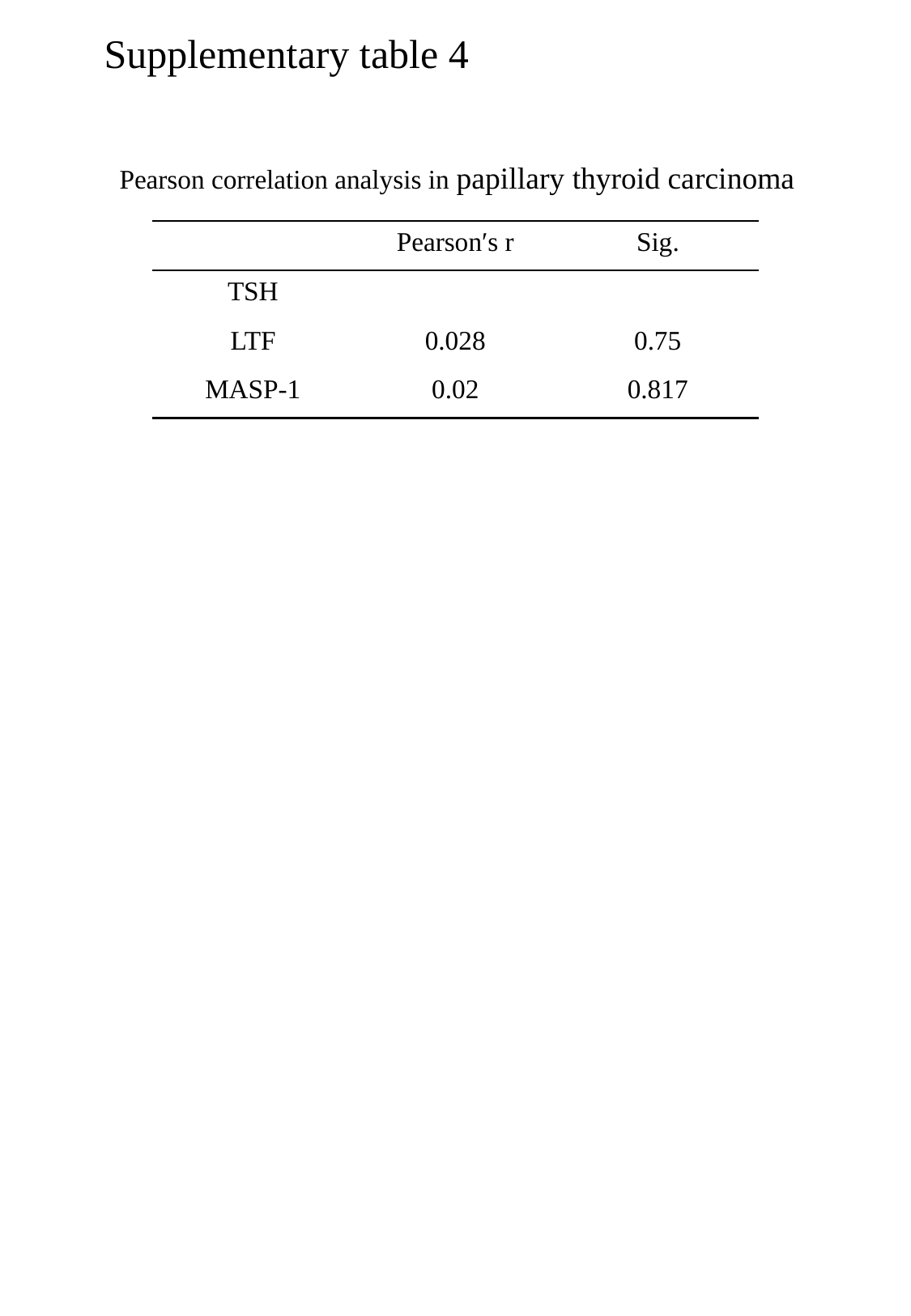

Supplementary table 4
Pearson correlation analysis in papillary thyroid carcinoma
| | Pearson′s r | Sig. |
| --- | --- | --- |
| TSH | | |
| LTF | 0.028 | 0.75 |
| MASP-1 | 0.02 | 0.817 |
